# Hypertension among adolescents in sub-Saharan Africa: a systematic review

**DOI:** 10.3389/fcvm.2023.1251817

**Published:** 2023-12-07

**Authors:** Mussa K. Nsanya, Rachel Abramson, Godfrey A. Kisigo, Andy Hickner, Elias C. Nyanza, Robert N. Peck, Saidi H. Kapiga

**Affiliations:** ^1^ Mwanza Intervention Trials Unit/National Institute for Medical Research, Mwanza, Tanzania; ^2^ Weill Cornell Medicine – Cornell University, New York, NY, United States; ^3^ Department of Infectious Disease Epidemiology, London School of Hygiene and Tropical Medicine, London, United Kingdom; ^4^ Catholic University of Health and Allied Sciences, School of Public Health, Mwanza, Tanzania

**Keywords:** hypertension, blood pressure, risk factors, adolescents, sub-Saharan Africa, cardiovascular complications

## Abstract

**Introduction:**

Hypertension (HTN) among adolescents is common in high-income countries, and leads to increased premature cardiovascular diseases (CVD). In sub-Saharan Africa (SSA), the prevalence of HTN among adolescents, associated risk factors and CVD complications are not well-described. Such data is needed for planning public health programs to prevent premature CVD in SSA.

**Methods:**

We systematically searched 5 databases (MEDLINE, Embase, Google Scholar, Web of Science, and African Index Medicus) from their establishment to December 2021. Key search terms were: adolescent, arterial hypertension, and names of the 48 countries in SSA. We used Covidence® to manage the search results. The review was registered in the Open Science Framework (OSF) https://osf.io/p5sbt/.

**Results:**

We identified 4,008 articles out of which we screened 3,088 abstracts, and reviewed 583 full-text articles. We finally included 92 articles that were published between 1968 to December 2021. The majority were cross-sectional studies (80%) and conducted in school settings (78%). The risk of bias was low for 59 studies (64.1%), moderate for 29 studies (31.5%), and high for 4 studies (4.3%). Overall, the prevalence of HTN varied widely from 0.18% to 34.0% with a median (IQR) of 5.5% (3.1%, 11.1%). It was relatively higher in studies using automated blood pressure (BP) devices, and in studies defining HTN using thresholds based on percentile BP distribution for one's height, age, and sex. In addition, the prevalence of HTN was significantly higher in studies from Southern Africa region of SSA and positively correlated with the year of publication. Across studies, traditional risk factors such as age, sex, body mass index, and physical inactivity, were commonly found to be associated with HTN. In contrast, non-traditional risk factors related to poverty and tropical diseases were rarely assessed. Only three studies investigated the CVD complications related to HTN in the study population.

**Conclusion:**

The prevalence of HTN among adolescents in SSA is high indicating that this is a major health problem. Data on non-traditional risk factors and complications are scarce. Longitudinal studies are needed to clearly define the rates, causes, and complications of HTN.

**Systematic Review Registration:**

https://osf.io/p5sbt/, identifier (10.17605/OSF.IO/P5SBT).

## Introduction

Hypertension (HTN) among adolescents is common globally, but most data come from high-income countries (1). Much less information is available from sub–Saharan Africa (SSA) where adolescents constitute about 18% of the population (2). In high-income countries, obesity is the major risk factor for HTN, and other traditional risk factors such as physical inactivity, unhealthy diet, and family history of HTN are also common (3, 4). In SSA, potential risk factors related to poverty and tropical diseases, also referred to as “non-traditional” risk factors, may be common. They include low birth weight, malnutrition, stunted growth, malaria, and/or other tropical infectious diseases. However, there is limited information describing the contribution of non-traditional risk factors for HTN among adolescents in Africa (3, 5–9).

Increasing evidence from long-term follow-up studies in high-income countries shows that HTN during adolescence is strongly associated with premature onset of cardiovascular diseases (CVD) including left ventricular hypertrophy, chronic kidney disease, decline in cognitive function, and type-2-diabetes mellitus (10–13). By comparison, little is known about blood pressure (BP) trajectory and its potential complications among adolescents in SSA (14, 15).

We are aware of three systematic reviews (and meta-analyses) describing the burden of HTN in children and adolescents in Africa (16–18). The reviews used published data from 1996 to 2017 and from 2017 to 2020, and from 2010 to 2021with the pooled estimates for prevalence of HTN of 5.5% and 7.5% and 9.9% respectively. The major limitations of two of these reviews were that they combined BP measurements for adolescents with those of children as young as 2 years. Also, neither of the reviews reported non-traditional risk factors and complications of HTN. Since the epidemiology of HTN varies between adolescents and children, studies that focus on adolescents only may provide useful information specific for this population.

Therefore, the current systematic review aims to describe the burden, risk factors, and complications of HTN among adolescents aged 10–19 years in SSA. Findings from this study will inform (1) programs promoting regular and proper BP measurements among adolescents; (2) primary CVD preventive interventions targeting adolescents; and (3) the design of new studies aimed to bridge existing gaps in the literature.

## Methods

### Databases and search strategy

We systematically searched for studies on HTN among adolescents aged 10–19 years in SSA, published in any language, from a range of databases from their inception until December 2021. Two investigators (MKN and RHA) were supported by a librarian (AH) to develop and conduct the search strategy. We searched MEDLINE, Embase, and African Index Medicus to identify relevant published literature. We searched Embase and Web of Science Core collection to identify studies that had been presented as conference abstracts but not yet published as full-length journal articles. We searched Google Scholar to identify potentially relevant information apart from the published research articles (19). The search strategy consisted of three concepts, combined with Boolean operators: adolescents, sub-Saharan Africa (names of the 48 countries in SSA), and hypertension. Both controlled vocabulary and free-text keywords were identified using the Yale MeSH Analyzer (20). Proximity operators were incorporated to retrieve variations of phrases. No limits were used for any of the databases. Terms used for HTN were “arterial hypertension”, “hypertension” and “blood pressure”. Terms for adolescent were “adolescent”, “teen”, and “youth”. The specific search strategies used for each of the databases are attached in Supplementary Appendix 1.

### Selection criteria

Using Covidence (Melbourne, VIC 3000, Australia), two investigators (MKN and RHA) independently assessed the articles for eligibility in two steps. First, they independently screened the titles and abstracts of the retrieved articles. Then, they obtained full texts of potentially eligible articles and independently reviewed them for possible inclusion. A third reviewer (GAK) resolved any disagreements between the two investigators. Throughout this selection process, duplicates were removed both by Covidence and the two reviewers. We used Google Translate for studies published in languages other than English.

We included studies if they were: observational (cross-sectional, case-control, and cohort studies) or experimental studies, enrolled adolescents residing in SSA, reported at least one of the outcomes of interest (incidence/prevalence, risk factors, and complications of HTN), and reported data for a group or subgroup of participants within the ages of 10–19 years.

We excluded studies if they were animal studies, only reported non-systemic HTN (e.g., portal hypertension, pulmonary hypertension, and intracranial hypertension), case studies, case series, studies with sample size less than 5, qualitative studies and systematic reviews, studies including only participants with high BP, studies including only adolescents from a single high-risk group (e.g., those with HIV, sickle cell disease, malnutrition), and studies including adolescents with secondary HTN.

### Data extraction procedures

The two investigators (MKN and RHA) independently extracted data using a pre-validated data extraction table. This process did not use any automation tools. Extracted data included: the author's name, year of publication, country of study, study design, participant's age range, average age, sample size, type of BP measurement device used (automated vs. manual), criteria used for defining HTN, prevalence and/or incidence estimates, risk factors and complication of HTN. We only extracted data for the eligible age range. We hand-calculated proportion of adolescents with HTN in the eligible age range if this information was not reported and where the numerator and the corresponding denominator were available. For cohort studies, we used prevalence estimates from baseline data and incidence from follow-up visits. We left blank any information which was either missing or not reported for the eligible age range. A third reviewer (GAK) resolved any discrepancies in the extracted data.

### Evaluation of risk of bias

The two investigators (MKN and RHA) independently assessed each of the included studies for risk of bias using the Newcastle-Ottawa Scale (NOS) for assessment of the quality of non-randomized studies (21). The assessment was done on three domains: (1) selection of study participants, (2) comparability of participants in different outcome groups, and (3) ascertainment of study outcomes or exposure status. Across the three domains, a range of “high-quality” items could score a star (depending on the study design). Case-control and cohort studies have a total of 8 items with a maximum score of 9 stars. And cross-sectional studies have a total of 7 items with a maximum score of 10 stars. Studies with a total score of ≥7 stars were regarded as of high quality, 4–6 stars as of moderate quality, and 0–3 stars as of low quality (high risk of bias). We resolved any disagreements on the assessment of the risk of bias by consensus.

### Data synthesis and analysis

We summarized the study selection process using a PRISMA flow diagram (22). We used tables to show characteristics of included studies, prevalence and/or incidence of HTN, and associated risk factors. We summarized our findings using proportions (for categorical data), and mean and/or median (for continuous data). We assessed the association between prevalence of HTN and each of the following study characteristics: year of publication, study settings (rural, rural/urban and urban), sub Saharan Africa region from which the studies came from (West, South, East, and Central), type of BP measurement device used (manual vs. automated), and criteria for defining HTN (cut offs based on a fixed vs. percentile BP values). We used meta-regression analysis to assess the adjusted association between prevalence of HTN and all the five study characteristics mentioned above. We did not perform pooled analysis due to heterogeneity in methods as well settings/context across the studies.

#### Ethical considerations

This study was exempted from ethical approval as it involved a review of published data. To our knowledge, all included studies obtained ethics clearance before data collection. The protocol for this review was registered in the Open Science Framework (OSF) https://osf.io/p5sbt/.

#### Role of funding organization

The funders had no role in the study design, data collection, data analysis, data interpretation, and writing of this manuscript.

## Results

**Figure 1:**
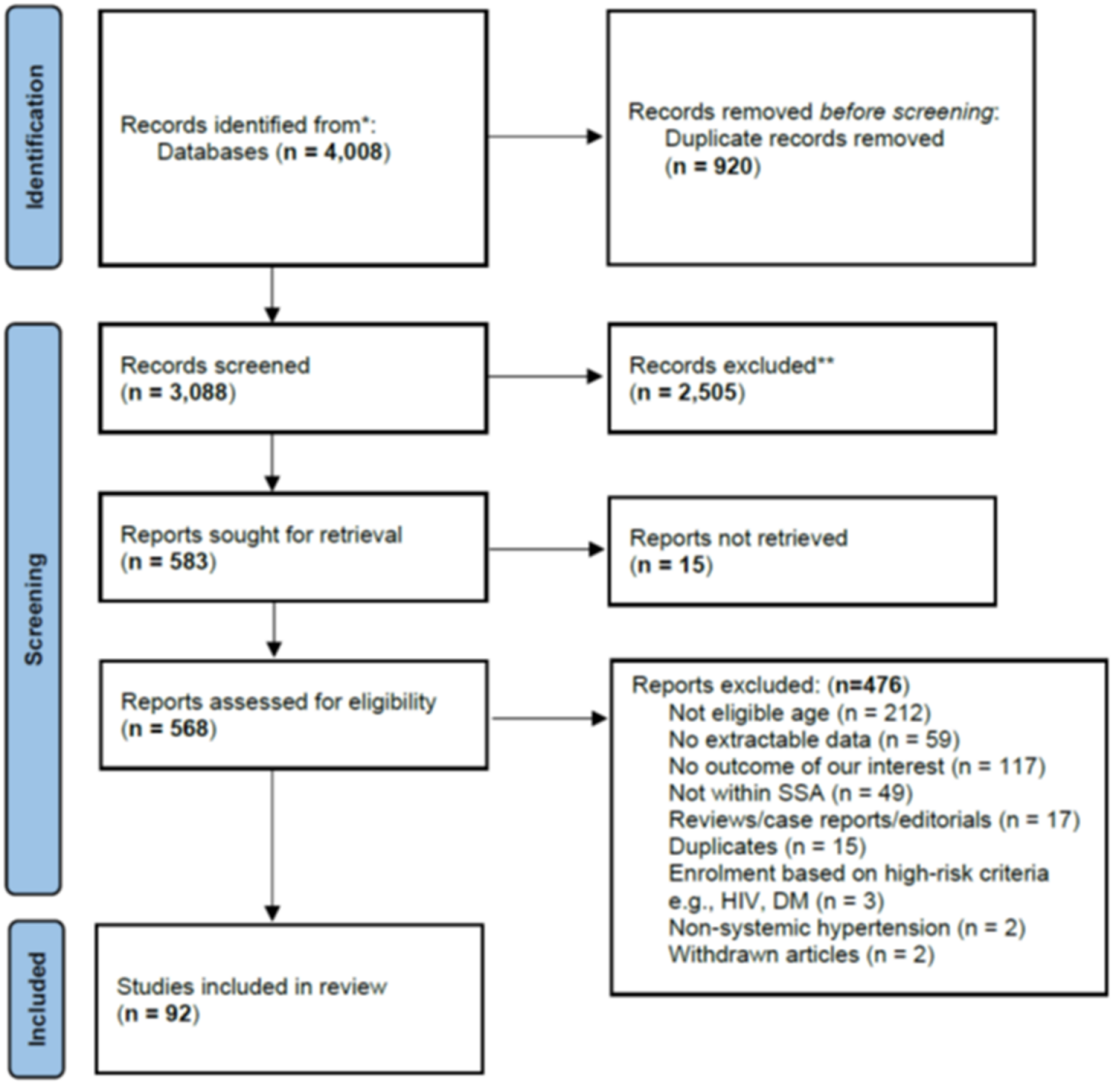
PRISMA flow diagram showing the study selection process.

We identified 4,008 articles, and after removing 920 duplicates, we screened the title and abstract of the remaining 3,088 articles and selected 583 articles for full-text review. We could not retrieve the full texts of 15 publications (Supplementary Appendix 2). Of the remaining 568 articles, 476 were excluded after a full-text review (their exclusion reasons are summarized in the Figure 1 PRISMA flow diagram below. We finally included 92 articles in our systematic review.

### Characteristics of the included studies

Table 1 shows the characteristics of the 92 included studies. The studies were published from 1968 to 2021, with the majority (75%) published between 2010 and 2021. The studies came from 14 (29%) out of the 48 countries in SSA. The majority of the studies came from the west (46.2%), followed by the south (29.7%), east (22.0%), and central (2.2%) African regions. Of the 92 studies, the majority were cross-sectional (80.0%), and conducted in schools (70.3%) and in urban settings (65.9%). They included a total of 89,599 subjects with sample sizes ranging from 53 to 7,746 (median = 670, IQR: 375, 1,080). The risk of bias was low for 59 studies (64.1%), moderate for 29 studies (31.5%), and high for 4 studies (4.3%).

**Table 1:** Characteristics of studies with data on hypertension among adolescents aged 10–19 years in sub-Saharan Africa (*N* = 92).

Author	Year of publication	Country	Study design	Age range	Average age	Sample size	Female (%)	Setting	Urban/Rural
Akinkugbe OO (23)	1968	Nigeria	Cross-sectional	10–19		1,119	48.8	Community	Rural
Johnson TO (24)	1971	Nigeria	Cross-sectional	10–19		370	59.5	Community	Urban
Akinkugbe A (25)	1976	Nigeria	Cross-sectional	15–19		103	100	Community	Rural
Ayobanji Ayoola E (26)	1979	Nigeria	Cross-sectional	11–19		487	39.6	School	Urban
Abu-Bakare A (27)	1983	Nigeria	Cross-sectional	11–18	14.3	684	100	School	Urban
Idahosa PE (28)	1985	Nigeria	Cross-sectional	15–19		109	0	Community	Urban
Eferakeya AE (29)	1986	Nigeria	Cross-sectional	10–15	12.5	679	45.5	School	Urban & Rural
Adams-Campbell LL (30)	1987	Nigeria	Cross-sectional	12–17		118	34.8	School	Urban
Kitange HM (31)	1993	Tanzania	Cross-sectional	15–19	16.8	1,673	55.2	Community	Urban & Rural
Muraguri PW (32)	1997	Kenya	Cross-sectional	13–18		403	49.4	School	Urban
Kane A (33)	1998	Senegal	Cross-sectional	12–19		132		Community	Rural
Longo-Mbenza B (34)	1999	Zaire	Cross-sectional	13–16		471	33.1	School	Urban & Semi-Urban
Schutte AE (#167) (35)	2003	South Africa	Cross-sectional	10–19		631	53.1	School	Urban & Rural
Schutte AE (#168) (36)	2003	South Africa	Cross-sectional	10–15		695	53.8	School	Urban & Rural
Schutte AE (#1885) (35)	2003	South Africa	Cross-sectional	10–15		631	53.1	School	Urban & Rural
VanRooyen JM (37)	2005	South Africa	Cross-sectional	10–15	12.4	775	53.1	School	Urban & Rural
Chiolero A (38)	2007	Seychelles	Cross-sectional	12–15		7,746	51.4	School	
Thurston SW (39)	2007	Seychelles	Cohort	12–15		524	53.4	Community	Urban
Mijinyawa MS (39)	2008	Nigeria	Cross-sectional	13–19	15.9	968	50.8	School	Urban
Monyeki KD (40)	2008	South Africa	Cross-sectional	10–13		1,307	49.3	School	Rural
Cournil A (41)	2009	Senegal	Cohort	16–19	17.9	1,288	46.4	Community	
Mijinyawa MS (42)	2009	Nigeria	Cross-sectional	13–18	15.6	718	56.9	School	Urban
Odey F (43)	2009	Nigeria	Cross-sectional	10–18	14.6	375	61	School	Urban
Ansa VO (44, 45)	2010	Nigeria	Cross-sectional	10–17	13.4	964	51	School	Urban
Ejike C (45)	2010	Nigeria	Cross-sectional	13–18	15.2	843	47.3	School	Urban & Semi-Urban
Kruger HS (46)	2010	South Africa	Cross-sectional	13–18	15.6	178	60.9	School	
Chiolero A (47)	2011	Seychelles	Cohort	12–15	12.5	3,461	51.8	School	
Hawkesworth S (48)	2011	Gambia	Interventional: randomized trial	11–17	14.1	1,267	48.2	Community	Rural
Mamabolo RL (49)	2011	South Africa	Cross-sectional	12–19		307	56.7	School	
Meehan KA (50)	2011	Sierra Leone	Cross-sectional	15–16		407		Hospital	Urban
Bukabau JB (51)	2012	DRC/Zaire	Cross-sectional	15–20	18.7	524	49.8	School	Urban
Griffiths PL (52)	2012	South Africa	Cohort	16	16	358	47	Community	Urban
Okoh BA (53)	2012	Nigeria	Cross-sectional	10–12		483	56.7	School	Urban
Okpere AN (54)	2012	Nigeria	Cross-sectional	10–19	13.6	615	51.4	School	Urban
Oyewole OO (55)	2012	Nigeria	Cross-sectional	12–18	14.9	1638	51.7	School	Urban
Goon D (56)	2013	South Africa	Cross-sectional	10–13		140	45.7	School	Rural
Lyngdoh T (57)	2013	Seychelles	Cohort	12–15		407	54.1	School	Urban
Okpere AN (58)	2013	Nigeria	Cross-sectional	10–19		820	49.9	School	Urban
Ujunwa FA (59)	2013	Nigeria	Cross-sectional	10–18	15	2,694	52	School	Urban
Mushengezi B (60)	2014	Tanzania	Cross-sectional	12–19	16.5	582	47.9	School	Urban
Okagua J (61)	2014	Nigeria	Cross-sectional	10–18	14.5	1,080	49	School	Urban
Kagura J (14)	2015	South Africa	Cohort	18	18	1,587	51.7	Community	Urban
Nkeh-Chungag B (62)	2015	South Africa	Cross-sectional	13–17	15.1	392	69.9	School	Semi-Urban
Odunaiya NA (63)	2015	Nigeria	Cross-sectional	15–18	16.4	1,079	53.5	School	Rural
Oyeyemi AY (64)	2015	Nigeria	Cross-sectional	13–18	15.6	1,048	37.7	School	Urban
Ratovoson R (65)	2015	Madagascar	Cross-sectional	15–17		638		Community	Urban & Rural
Awotidebe A (66)	2016	South Africa	Cross-sectional	13–14	13.9	310	61.9	School	
Munthali RJ (67)	2016	South Africa	Cohort			1,589	51.7	Community	Urban
Strassman BI (68)	2016	Mali	Cohort						
Uwaezuoke SN (69)	2016	Nigeria	Cross-sectional	10–19	14.8	2,419	48.7	School	Urban
Alicke M (70)	2017	Ghana	Cross-sectional	14–15	15.2	188	50	Hospital	Rural
Bedu-Addo G (6)	2017	Ghana	Cohort	14–15	14.8	155	47	Community	Rural
Etyang AO (71)	2017	Kenya	Cross-sectional	11–17	13.3	686	55.7	Community	Urban
Etyang AO (72)	2018	Kenya	Cross-sectional	11–17	13.3	609	55.5	Community	Urban
Ezeudu CE (73)	2018	Nigeria	Cross-sectional	10–19	14.6	984	52.2	School	Rural
Hendry LM (74)	2018	South Africa	Cohort		17.9	1,947	73.3	Community	Urban
Isezuo KO (75)	2018	Nigeria	Cross-sectional	10–18	14.5	800	47	School	Urban
Leyvraz M (76)	2018	Seychelles	Cohort	12–15	15.6	5,967	51.4	School	Urban & Rural
Masocha V (77)	2018	South Africa	Cohort	14	14.9	289	59.9	School	Urban
Nakiriba R (78)	2018	Uganda	Cross-sectional	12–18	15.4	688	100	School	Urban
Omisore AG (79)	2018	Nigeria	Cross-sectional	10–19	13.7	1,000	49	School	Urban & Rural
Abu OO (#201) (80)	2019	Nigeria	Cross-sectional	10–19	14	420	57.4	School	Urban
Adeomi AA (81)	2019	Nigeria	Cross-sectional	10–19	14.4	313	58.5	School	Urban
Amponsem-Boateng C (82)	2019	Ghana	Cross-sectional	15–17		571	61.2	School	
Chungag A (83)	2019	South Africa	Cross-sectional	10–14	11.9	540	53.7	School	
Frigati L (84)	2019	South Africa	Case-control	9–14	12	620	49.8	Community	Urban
Lule SA (#101) (85)	2019	Uganda	Cohort	10–11	10.2	1,119	47.9	Community	Urban & Rural
Lule SA (#3098) (86)	2019	Uganda	Cohort	10–11	10.4	815	49	Community	Urban & Rural
Lule SA (#409) (87)	2019	Uganda	Cohort	10–11	10.2	1,119	47.9	Community	Urban & Rural
Nkwana MR (88)	2019	South Africa	Cross-sectional	11–15		591	46.7	School	Urban
Nsanya MK (89)	2019	Tanzania, Uganda	Cross-sectional	12–15		827	50.4	School	Urban
Abiodun O (90)	2019	Nigeria	Cross-sectional	15–19	16.51	6,980	56.2	School	
Azupogo F (91)	2020	Ghana	Cross-sectional	15–19	16.9	1,727	49.6	Community	Urban & Rural
Katamba G (92)	2020	Uganda	Cross-sectional	12–19	15.6	616	65.6	School	Urban
Masocha V (93)	2020	South Africa	Cohort	14–16	14.9	186	56.5	School	Urban
Mokgwathi M (94)	2020	Botswana	Cross-sectional		17.1	252	68.3	School	Urban & Rural
Raphadu TT (95)	2020	South Africa	Cross-sectional	13–19		218	55.5	School	Urban & Rural
Sungwa EE (96)	2020	Tanzania	Cross-sectional	10–16		350		School	Urban
Ugochukwu EF (97)	2020	Nigeria	Cross-sectional	10–17		593	51.2	School	Urban
Ukoh U (98)	2020	Nigeria	Cross-sectional	10–19	15.1	2,401	50.2	School	Urban
Akinbodewa AA (99)	2021	Nigeria	Cross-sectional	10–17	12.9	53	52.8	Community	Rural
Ayogu RN (100)	2021	Nigeria	Cross-sectional	10–19		401	53.1	Community	Rural
Chungag A (101)	2021	South Africa	Cohort	10–14		411	54.3	School	Urban & Rural
DuPlessis JP (102)	2021	South Africa	Cross-sectional	13–14	15.2	172	61	School	Urban
Engwa G (103)	2021	South Africa	Cross-sectional	13–16		234		School	Rural
Letswalo BP (104)	2021	South Africa	Cross-sectional	13–16	14.1	232	78.4	School	Rural
Lwabukuna WC (105)	2021	Tanzania	Cross-sectional	14–19	16.9	217	68	School	Urban
Meer R (106)	2021	South Africa	Cohort	5–18		1,891	51.7	Community	Urban
Nganou-Gnindjio CN (107)	2021	Cameroon	Cross-sectional	12–19		1,392		School	Urban & Semi-Urban
Nsanya MK (4)	2021	Tanzania	Cross-sectional	11–15	13.9	500	56.6	School	Urban
Sekokotla AM (108)	2021	South Africa	Case-control	13–17		76	100	School	Urban
Shokunbi OS (109)	2021	Nigeria	Cross-sectional	10–19		488	64.3	School	Urban

### Methods of BP measurements

The studies used different devices for BP measurements, and the definition of HTN was not consistent across studies (Table 2).

**Table 2:** Studies that reported prevalence and incidence of hypertension among adolescents aged 10–19 years in sub-Saharan Africa (*N* = 78).

Author	Year of publication	Device for BP meas.	Days for BP meas.	Number of BP meas.	Calculation of final BP meas.	HTN definition or guideline	Pre-HTN (%)	HTN (%)	Elevated BP (%)	Incidence	Mean SBP (SD)	Mean DBP (SD)
Akinkugbe OO (23)	1968	Mercury	1	1		WHO, BP ≥ 140/90		2.8			106.4	65.6
Johnson TO (24)	1971	Mercury	1	1		WHO, BP ≥ 160/95		0.8			117.9	74.6
Ayobanji Ayoola E (26)	1979	Mercury	1	3	Average	BP > + 2 SD			3.3		106.5 (11.8)	61 (9)
Abu-Bakare A (27)	1983					WHO, BP ≥ 133/90		6.8			110.6 (11.1)	69.5 (10.5)
Idahosa PE (28)	1985	Automated	1	2	Average	WHO, BP ≥ 160/95	11.9	1.8			122 (15.7)	63 (11.1)
Eferakeya AE (29)	1986	Finapres	1	Continuous 5 min	Average	BP > + 2 SDs		4.47				
Adams-Campbell LL (30)	1987	Mercury	1	3	Average of 2nd & 3rd	WHO, BP ≥ 140/90		2.3				
Kitange HM (31)	1993	Mercury	1	2	Average	WHO, BP ≥ 160/95		0.4			114.1 (12.4)	66.1 (9.5)
Muraguri PW (32)	1997	Mercury	1	3	Average	Second Report		1			109.5 (9.9)	66.5 (6.5)
Kane A (33)	1998	Aneroid	1	1		Third Report		1.5			108.9 (12.2)	64.3 (10.1)
Schutte AE (35) (#167)	2003	Finapres	1	Continous	Average	Third Report			20.7			
Schutte AE (36) (#168)	2003	Finapres	1	Continous	Average	Third Report		17.3	20.8			
Schutte AE (35) (#1885)	2003	Finapres	1	Continous	Average	Third Report		15.5	18.4			
Chiolero A (38)	2007	Automated	1	2	Average	Fourth Report					110.5 (12)	67.2 (8.5)
Monyeki KD (40)	2008	Automated	1	3	Average	Fourth Report		4.9				
Cournil A (41)	2009	Mercury	1	3		WHO, BP ≥ 140/90		12.2			120.5 (12.8)	72.5 (12.4)
Mijinyawa MS (42)	2009	Mercury	1	3	Average	Third Report		4.5			111 (13.8)	73 (9.5)
Odey F (43)	2009	Mercury	1	3	Average	Fourth Report	7.5	6.7			114.6 (12.6)	62.9 (8.7)
Ansa VO (44)	2010	Mercury	1	2	Average	Fourth Report		1.8				
Ejike C (45)	2010	Mercury	1	2	2nd reading	BP > mean + 2SD	23.4	10.1				
Kruger HS (46)	2010	Mercury	1	2	Average	Fourth Report			26.4			
Chiolero A (47)	2011	Automated	1	2	Average	Fourth Report		7.3			109.8 (11.4)	66.8 (8.2)
Hawkesworth S (74)	2011	Automated	1	3		Third Report		5.46			110.5 (9.0)	64.7 (7.6)
Meehan KA (50)	2011	Mercury	1	1		WHO-ISH 1999, JNC7		1.2			104.2 (12.3)	64.3 (8.4)
Bukabau JB (51)	2012	Aneroid	1	3	Average	WHO, BP ≥ 140/90		3.1			107.3 (11.3)	70.9 (8.1)
Griffiths PL (52)	2012	Automated	1	3	Average of 2nd & 3rd	Fourth Report	11.2	1.9			114.8 (10.4)	
Okoh BA (53)	2012	Mercury	1	3	Average	Fourth Report		3.9				
Okpere AN (54)	2012	Mercury	1	3	Average	Second Report		2.8				
Oyewole OO (55)	2012	Aneroid	1	1		Fourth Report & JNC7	1.5	0.18			81.4 (14.3)	48.5 (9.4)
Goon D (56)	2013	Automated	1			Fourth Report			0.6		99.4 (9.4)	54.8 (5.7)
Okpere AN (58)	2013	Mercury	1	3	Average	Fourth Report		3.2			114.2 (9.3)	70.4 (7.8)
Ujunwa FA (59)	2013	Mercury	3	3	Average	Fourth Report	13.5	12			108.31 (11.8)	71.2 (7.9)
Mushengezi B (60)	2014	Automated	1	2	Average	Fourth Report & WHO		4.0			120 (11)	69 (8)
Okagua J (61)	2014	Mercury	1	3	Average	Fourth Report		4.3			111.1 (14.7)	66.3 (11.7)
Kagura J (14)	2015	Automated	1	3	Average of 2nd & 3rd	Fourth Report	12.2	15.7			117.9 (11.0)	71.0 (9.0)
Nkeh-Chungag B (62)	2015	Automated	1	3	Average	Fourth Report	12.3	21.2			119.5 (1.5)	72.0 (1)
Oyeyemi AY (64)	2015	Mercury	1	3	Average of 2nd & 3rd	WHO, BP ≥ 125/80	11.2	2.4				
Ratovoson R (65)	2015	Automated	1	2	Average	JNC 7		7.4				
Awotidebe A (66)	2016	Mercury	1	3	Average of 2nd & 3rd	Fourth Report	8.7	4.3			105.3 (11.4)	68.6 (8.9)
Munthali RJ (67)	2016	Automated	1	2	Average	Fourth Report & JNC7			34.9			
Uwaezuoke SN (69)	2016	Mercury	1	3	Average	Fourth Report & JNC7		10.7				
Alicke M (70)	2017	Automated	1	3	Average of 2nd & 3rd	Fourth Report		9			110	68
Bedu-Addo G (6)	2017	Automated	1	3	Average of 2nd & 3rd	Fourth Report		10			110 (11)	68 (9)
Etyang AO (71)	2017	Automated	1	3	Average of 2nd & 3rd	AHA 2014		4.2			117 (12)	64 (8)
Etyang AO (72)	2018	Automated + 24 h ABPM	2	3	Average of 2nd & 3rd	AHA 2014		3.9			117 (11)	64 (7)
Ezeudu CE (73)	2018	Automated	2	3	Average of 2nd & 3rd	Fourth Report		6.3			110.5 (10.2)	71.5 (8.5)
Isezuo KO (75)	2018	Mercury	3	3	Third measure	Fourth Report	7.5	3.1			111.7 (13.2)	67.7 (9.6)
Leyvraz M (76)	2018	Automated	1	2	Average	Fourth Report			8.5		111.6 (11.7)	68.4 (8.5)
Masocha V (77)	2018	Automated	1	2	Average	IDF			9.7	5% over 3 years	103.2 (9.8)	67.7 (8.0)
Nakiriba R (78)	2018	Automated	1	3	Average			11.6				
Omisore AG (79)	2018	Mercury	1	2	Average	Fourth Report		4.1			102.9 (13.5)	66.0 (9.9)
Abu OO (80) (#201)	2019		1			Fourth Report	8.8	6.9			104.8 (14.8)	67.1 (10.5)
Adeomi AA (81)	2019	Automated	1	2	Average	Fourth Report			32.9			
Amponsem-Boateng C (82)	2019	Automated & Mercury	1		Average	JNC 7	35	3.1				
Chungag A (83)	2019	Automated	1	3	Average	AAP 2017	12.2	20.7			111.5 (0.65)	71.9 (0.45)
Lule SA (#101) (85)	2019	Automated	1	3	Average of 2nd & 3rd	Fourth Report	10.5	8.4			105.9 (8.3)	65.2 (7.3)
Abiodun O (90)	2019	Mercury	1	3	Average of 2nd & 3rd	AAP 2017		25.3	25.1		118.6 (11.8)	68.3 (8.8)
Nkwana MR (88)	2019	Automated	1	2	Average			26.7			108.8 (13.5)	71.7 (11.3)
Nsanya MK (89)	2019	Automated	1	3	Average	Fourth Report	22.0	15.0	36.8			
Frigati L (84)	2019	Automated	1			Fourth Report		13.9			105.5	67.4
Azupogo F (91)	2020	Automated	1	3	Average of 2nd & 3rd	Fourth Report		0.2	20.4		110.5	68.5
Katamba G (92)	2020	Automated	1	3	Average of 2nd & 3rd	Fourth Report, ESH 2009, AHA 2017	7.1	3.1			113.3 (9)	66.5 (8.1)
Masocha V (93)	2020	Automated	1	2	Average	IDF	5				103.5 (10.5)	66.7 (8.4)
Mokgwathi M (94)	2020	Automated	2	2	Average	Fourth Report, JNC8	15.5	13.1			118 (13.2)	71.8 (9.5)
Raphadu TT (95)	2020	Automated	1	2	Average	Fourth Report	27.1	8.7				
Ugochukwu EF (97)	2020	Mercury	1	3	Average	Third Report	11.0	8.5			110.6 (12.6)	70.5 (8.5)
Ukoh U (98)	2020	Automated & Mercury	1	3	Average	Fourth Report & JNC 7		4.6			106.7 (11.4)	63.6 (7.3)
Sungwa EE (96)	2020	Automated	1	3	Average	Fourth Report			21.4		113.4	65.4
Akinbodewa AA (99)	2021	Mercury	1	1		Fourth Report	13.2	13.2			106.2 (15.5)	63.7 (10.7)
Ayogu RN (100)	2021	Automated	1	2	Average	AAP 2017	10.7	19				
Chungag A (101)	2021	Automated	1	3	Average	AAP 2017		5.1	32.8		111.8	72.1
DuPlessis JP (102)	2021	Finapres	1	Continuous	Average	AAP 2017	19	34			117 (13.3)	77 (9.2)
Letswalo BP (104)	2021	Automated	1	3	Average	Fourth Report	15.5	23.3			115.9 (9.0)	72.7 (6.3)
Lwabukuna WC (105)	2021	Mercury	1	2	Average	IDF		2.3			111.5 (8.7)	65.2 (5.9)
Meer R (106)	2021	Automated		3	Average	Fourth Report				57 cases/1000 pyrs		
Nganou-Gnindjio CN (107)	2021	Automated	1	3	Average	AAP 2017/JNC8		11.1				
Nsanya MK0 (4)	2021	Automated + 24 h ABPM	2	2	Average	AAP 2017			10.2			
Sekokotla AM (108)	2021	Automated	1	3	Average		11.8	19.7	31.6		117	71
Shokunbi OS (109)	2021	Mercury	1	3	Average	AAP 2017		10.5	19.3		110.6 (13.7)	71.9 (11.0)

Second Report: National Heart, Lung and Blood Institute (NHLBI) task force report on blood pressure control in children (1987), children and adolescents up to 18 years.

Third Report: National Heart, Lung and Blood Institute (NHLBI) update on the 1987 task force report on high blood pressure in children and adolescents up to 18 years.

Fourth Report: National Heart, Lung and Blood Institute (NHLBI) task force report on the diagnosis, evaluation, and treatment of high blood pressure in children and adolescents up to 17 years.

ESH 2009: European Society of Hypertension (ESH) on management of high blood pressure in children and adolescents up to 17 years.

AHA 2014: American Heart Association (AHA) update, A scientific statement on ambulatory BP monitoring in children and adolescents up to 17 years.

AAP 2017: American Academy of Paediatric—clinical practice guideline for screening and management of high blood pressure in children and adolescents up to 12 years.

JNC7: The Joint National Committee on prevention, detection, evaluation and treatment of high blood pressure “he seventh report”—(adults ≥18years).

JNC8: The Joint National Committee on prevention, detection, evaluation and treatment of high blood pressure “he eighth report”—(adults ≥18years).

WHO/ISH 1999: The World Health Organization—International Society of Hypertension Guidelines for Management of hypertension—(adults ≥18years).

IDF: The International Diabetes Federation (IDF) consensus definition metabolic syndrome—(adults ≥18years).

Of 79 studies shown in Table 2, 39 studies (49.4%) used automated BP machines, 33 studies (41.7%) used manual (mercury or aneroid) BP machines, 2 studies (2.6%) used both automated and manual BP machines, and 4 studies (5.1%) used Finapres (Finger artery pressure)—an automated device which records BP measurements “continuously” from a finger artery. Only 3 studies used 24-h ambulatory BP monitoring for confirming HTN (4, 71, 72).

Of the 79 studies, 71 (89.8%) had their BP measurements obtained in one day. Of those 71 studies, the number of BP measurement obtained in one day ranged from one in 6 studies (8.5%), two in 19 studies (26.8%) and three in 37 studies (52.1%). In addition, 5 studies (7.0%) collected more than 3 BP measurements *(“continuous”*), and this information was not reported in 4 studies (5.6%).

Of the 68 studies with more than one BP measurements, most studies 51(75.0%) used an average of all BP measurements to obtain final BP measure. In addition, 14 studies (20.5%) used an average of second and third BP measurements, 1 study (1.5%) only used second measurement, 1 study (1.5%) only used third measurement and this information was not reported in another 1 study (1.5%).

### Definitions of hypertension

Of the 77 studies with information on standard guidelines used for defining HTN (Table 2), 58 (75.3%) used one of the six American guidelines for the diagnosis and management of HTN in children and adolescents. The 2004 “*Fourth Report”* was the most commonly used version in 39 studies (50.6%) (110). In addition, the fixed BP cut-offs for defining HTN in adults were used in 10 studies (13.0%), often in older adolescents aged 18 years and above. Fewer studies used the z-distribution (*n* = 3) and the International Diabetes Federation definitions (*n* = 3).

Guidelines for defining HTN among adolescents have evolved over the years (Supplementary Appendix 3) provides a summary of the guidelines that were used for diagnosing HTN in the included studies. Overall, the aim for guideline revisions over the years was to account for age, height, and sex differences in BP thresholds for HTN. Also, in particular to American guidelines, the reference population size has increased from about 9,000 in 1977 to nearly 50,000 adolescents with normal BMI and percentile thresholds for HTN have decreased in the most recent guidelines (11).

### Prevalence and incidence of HTN

Of the 92 studies, 78 studies (84.6%) reported estimates for prevalence of pre-hypertension and/or elevated BP and/or HTN with only 2 studies reporting incidence of HTN (Table 2). The incidence of HTN was estimated at 5% over 2 years (77) and 57 cases per 1,000 person-years (106).

The prevalence of pre-hypertension was reported in 27 studies and ranged from 1.5% to 35%, with median (IQR) = 11.8% (8.7%, 15.5%). The prevalence of HTN was reported in 65 studies and ranged from 0.18% to 34.0%, with median (IQR) = 5.5% (3.1%, 11.1%); while the prevalence of elevated BP (combined pre-hypertension and HTN) was reported in 18 studies and ranged from 0.6% to 36.8%, and median (IQR) = 20.8% (10.2%, 31.6%).

### Prevalence of HTN across the four African regions in sub-Saharan Africa

Of all 65 studies with information on prevalence of HTN, studies from south African region (*n* = 13) had higher HTN prevalence, median (IQR) of 13.9% (5.1% to 21.2%), followed by west African region with a median (IQR) of 4.5% (2.6% to 9.5%), east African region with a median (IQR) of 4.2% (3.1% to 8.4%), and central African region with a median (IQR) of 3.1% (3.1% to 3.1%).

#### Factors associated with prevalence of HTN

**Figure 2:**
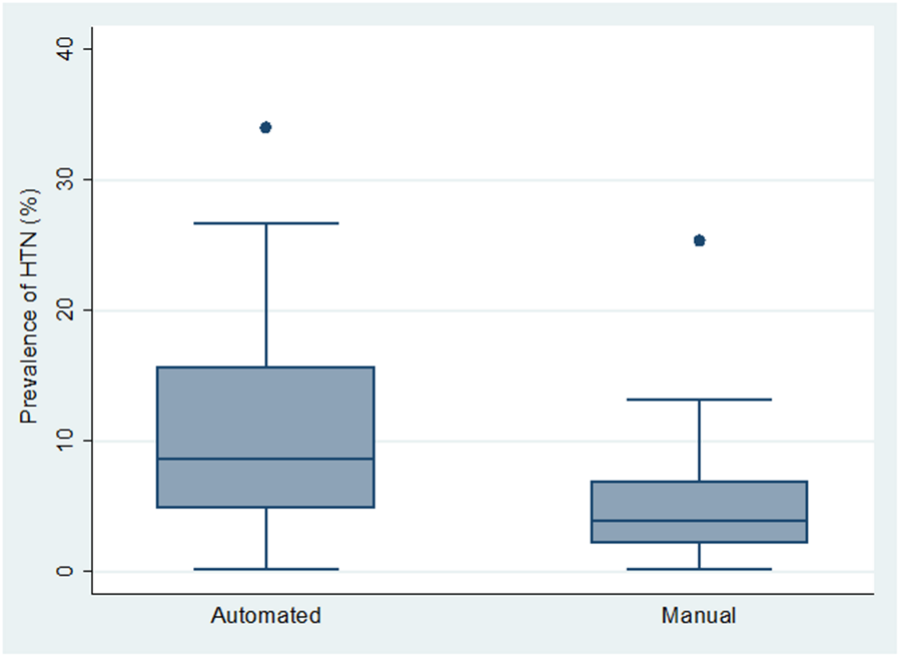
Box and whiskers plot showing the distribution of hypertension prevalence across the 2 types of BP measurement device.

Overall, studies in which BP measurements were obtained using automated BP devices (*n* = 31) had significantly higher mean HTN prevalence (11.1% (95% CI: 8.2%–14.1%)), than those using manual devices—Mercury or Aneroid BP machines (*n* = 31) (5.4% (95% CI, 3.6%–7.2%)) (*p* = 0.0006) (Figure 2).

**Figure 3:**
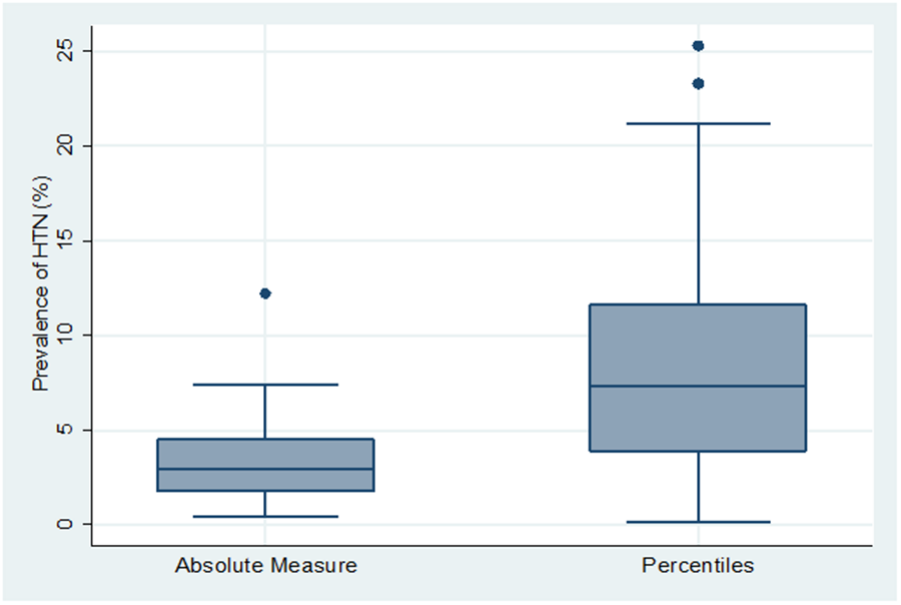
Box and whiskers plot showing the distribution of hypertension prevalence across 2 types of hypertension definition.

Similarly, studies in which HTN was defined using cut offs based on a percentile BP distribution according to age, sex, and height (*n* = 43) had significantly higher mean HTN prevalence (8.7% (95% CI: 6.7%–10.7%)), than those using cut-offs based a fixed BP measure (*n* = 14), (3.8% (95% CI: 1.9%–5.6%)) (*p* = 0.004) (Figure 3).

**Figure 4:**
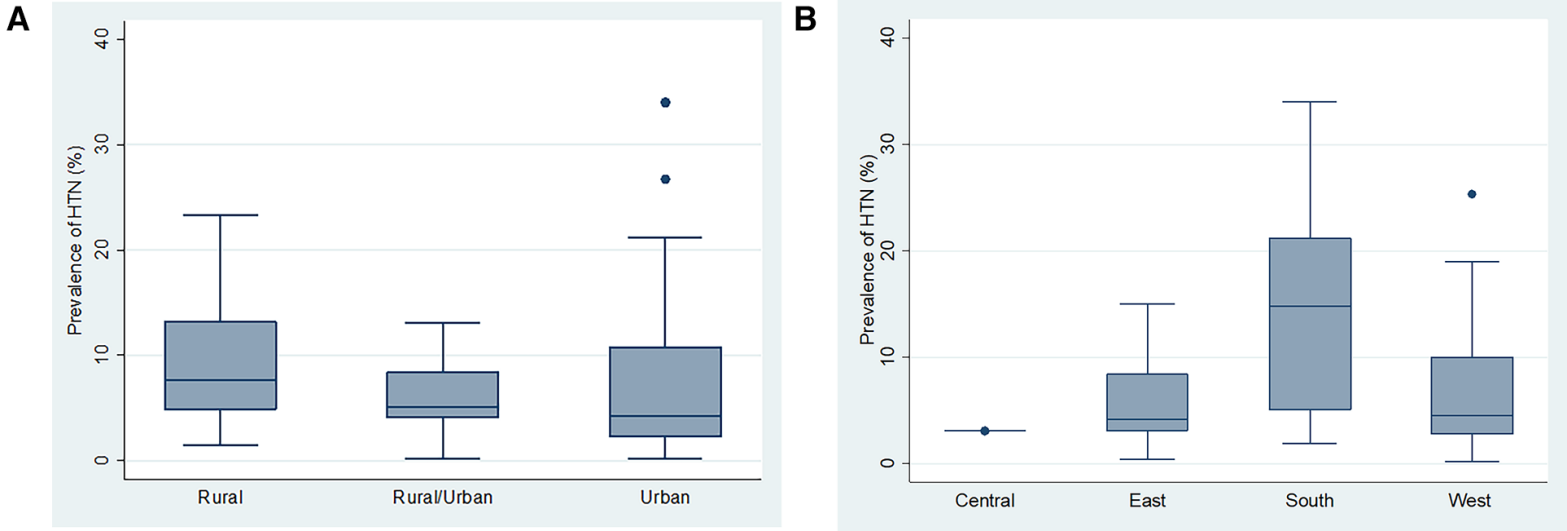
(**A**) Box and whiskers plot showing the distribution of hypertension prevalence across the study settings. (**B**) Box and whiskers plot showing the distribution of hypertension prevalence across the different SSA regions.

There were statistically significant differences between mean HTN prevalence across the four sub Saharan Africa regions (*F* = 7.49, *p* = 0.0002). However, there was no significant difference in mean HTN prevalence across study settings (rural, rural/urban and urban) (Figures 4A,B).

**Figure 5:**
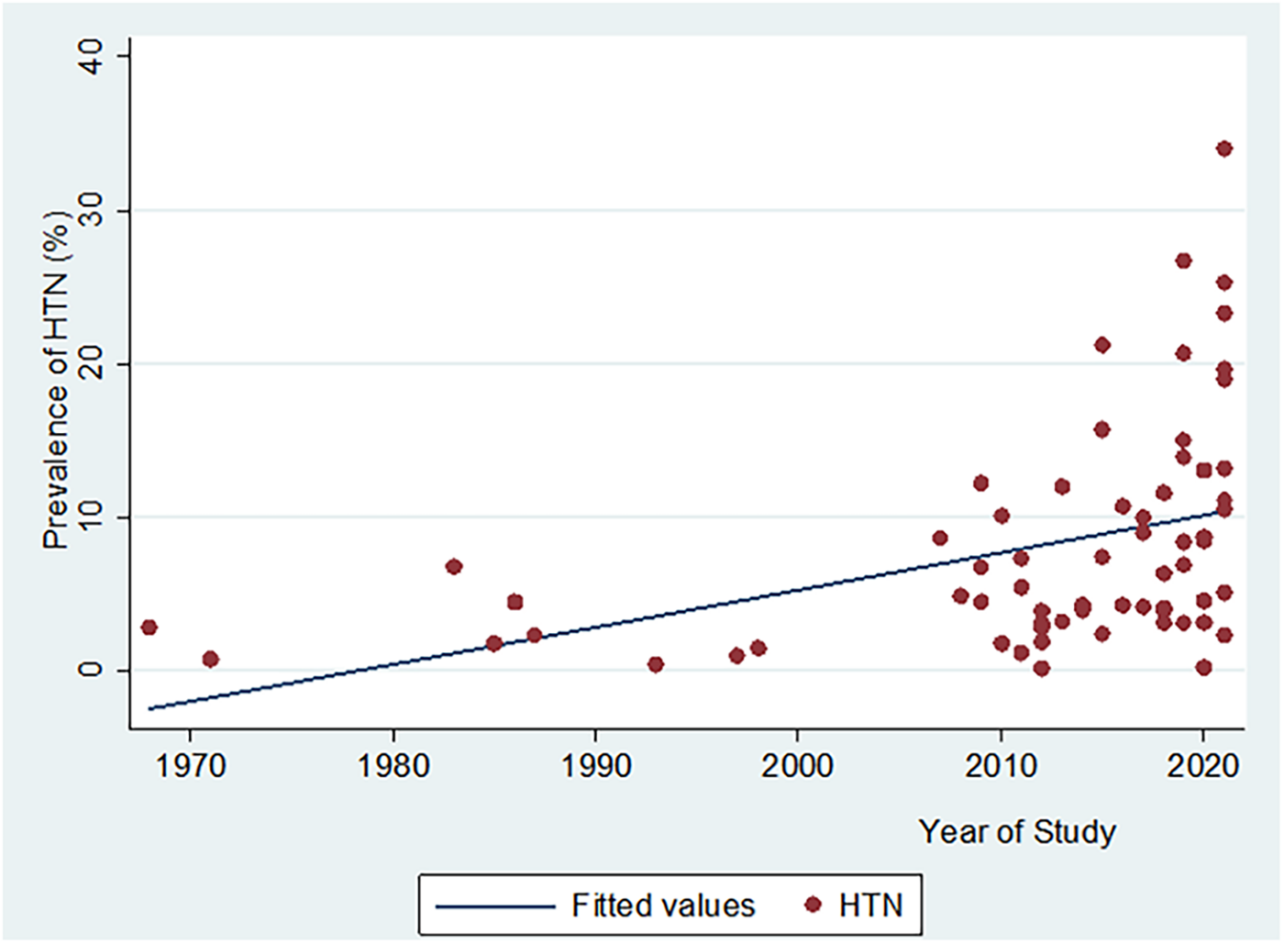
Scatter plot showing the relationship between hypertension prevalence and year of study publication.

There was a significant positive correlation between HTN prevalence, and year of study publication (*n* = 64, Pearson Correlation coefficient “*r*” = 0.4, *p* = 0.0009) (Figure 5).

In an adjusted meta regression analysis model (including year of publication, study settings, African region, type of BP measurement device used, and criteria for defining HTN), the year of publication (*β* = 0.14%, 95% CI: −0.003% to 0.28%, *p* = 0.05) and African regions (*F* = 3.95, *p* = 0.01) remained significantly associated with higher prevalence of hypertension. (Adjusted *R*^2^ = 0.38). Additionally, on average HTN prevalence estimates from Southern Africa were higher by 6.05%, 95% CI (1.97% to 10.13%, *p* = 0.005) than those from Western Africa.

#### Risk factors for hypertension

Table 3 summarizes common risk factors for HTN among adolescents in SSA. Of all 92 studies, 79 (85.9%) assessed at least one kind of traditional and/or non-traditional risk factor, and a majority (79.7%) were cross-sectional studies. Most of the reported risk factors were significantly associated with HTN although some of the associations were in an unexpected direction (82, 91, 100).

**Table 3:** Risk factors for hypertension among adolescents aged 10–19 years in sub-Saharan Africa.

Traditional risk factors		
Risk factor	With significant association	Without significant association
Body Mass Index (BMI) and other anthropometric measures {*n* = 60}	Overweight/Obesity {*n* = 23} (39, 45, 46, 52, 54, 57, 58, 71–73, 76, 77, 82, 85, 88, 90, 91, 100, 102, 104, 106, 108, 109) Higher BMI {*n* = 12} (38, 59, 62, 66, 69, 79, 81, 83, 89, 96, 97, 106) Waist circumference: Higher waist circumference {*n* = 6} (46, 66, 69, 83, 90, 104) Waist to height ratio: Higher waist to height ratio {*n* = 4} (66, 81, 90, 104) Waist to hip ratio: Higher waist to hip ratio {*n* = 1} (69) Neck circumference: increasing neck circumference {*n* = 1} (92) Thigh circumference: higher thigh circumference {*n* = 1} (104) Body fat percentage: higher body fat percentage {*n* = 2} (46, 66)	BMI {*n* = 9} (4, 32, 43, 63, 70, 92, 94, 98, 107) Stunted growth {*n* = 1} (37)
Sex {*n* = 53}	Female {*n* = 16} (39, 43, 58, 64, 70, 72, 73, 76, 83, 85, 91, 94, 97, 107, 109) Male {*n* = 9} (24, 71, 75, 89–92, 94, 95)	{*n* = 27} (24, 26, 30, 31, 36, 38, 41, 42, 45, 52–54, 60, 66, 75, 77, 79, 81, 88, 90, 95, 96, 99, 106, 108, 111)
Age {*n* = 45}	Older {*n* = 31} (31, 32, 37, 41, 43, 53, 63, 64, 70, 94, 98, 99, 107) Younger {*n* = 1} (100)	{*n* = 13} (24, 38, 45, 56, 66, 75, 81, 85, 90, 91, 95, 108)
Substance use/abuse {*n* = 8}	Cigarette smoking {*n* = 1} (94); Alcohol drinking {*n* = 4} (81, 92, 98, 100)	Cigarette smoking {*n* = 1} (59) Alcohol drinking {*n* = 1} (89)
Diet {*n* = 9}	Macronutrients: Deficiency in protein; Skipping meals daily (35, 100) Micronutrients: Deficiency in biotin B7, folic acid, pantothenic acid B5, zinc, vitamin & Energy; vitamin A, vitamin C, vitamin E, nicotinic acid, vitamin B12, biotin and phosphorus (35, 70) Sugar, salt and fat: Adding table salt; higher egg consumption; polyunsaturated fats; fried food; soft drinks; high sugar consumption > 10 teaspoons per day; eating fried/baked snacks (4, 96, 100, 109) Vegetables and fruits: low fruit and vegetable consumption; fibre (35, 89, 109)	
Physical inactivity {*n* = 7}	Physical inactivity {*n* = 4} (38, 77, 82, 97) Higher frequency of watching Television—protective among girls {*n* = 1} (91)	{*n* = 2} (4, 100)
Cardiometabolic risk factors {*n* = 7}	Cardiometabolic risks tend to cluster among females {*n* = 1} (70); lower eGFR {*n* = 1} (71); Higher pulse wave velocity {*n* = 2} (71, 104); Higher fasting glucose {*n* = 1} (104); high resting pulse >90bpm {*n* = 1} (4)	
Heredity {*n* = 8}	Genes—NOSIAP, MYRE, POC1B {*n* = 1} (74) Family history of hypertension {*n* = 5} (39, 54, 85, 92, 107)	Genes {*n* = 1} (86) Family history of hypertension {*n* = 1} (58)
Non-traditional risk factors		
Socioeconomic factors (*n* = 21)	Higher SES: Higher parental education {*n* = 2} (30, 85); higher socioeconomic class {*n* = 3} (42, 75, 98); parent income {*n* = 1} (64); Attending private school {*n* = 2} (73, 96); living in urban area {*n* = 2} (45, 79); owning land—protective {*n* = 1}; (91) Lower SES: Living in rural area {*n* = 1} (96); Lower socioeconomic status {*n* = 4} (43, 52, 91, 97); Environmental factor: Winter weather {*n* = 1} (101)	Parental education {*n* = 1} (64); parent occupation {*n* = 1} (64); higher socioeconomic class {*n* = 1} (29); Attending private school {*n* = 1} (58); lower social class {*n* = 1} (58); indoor particulate matter pollution {*n* = 1} (101); Living in rural area {*n* = 1} (101)
Maternal and Natal factors {*n* = 15}	Maternal factors: Maternal exposure to MeHg {*n* = 1} (111); Maternal hypertension {*n* = 1} (111); Malaria in pregnancy {*n* = 1} (6); Younger maternal age {20—29years}—protective {*n* = 1} (52); higher maternal gestation BMI {*n* = 1} (85); Natal factors: Low birth weight {*n* = 1} (34); Weight gain in first year of life {*n* = 1} (47); Postnatal and childhood weight gain {*n* = 2} (41, 87); Born post-term {*n* = 1} (52); High BP at young ages {*n* = 2} (14, 76);	Birth weight {*n* = 1} (47); Maternal supplementation with calcium and protein from second trimester {*n* = 1} (48); stunted growth {*n* = 1} (37)
Blood disorders {*n* = 5}	Higher haemoglobin concentration {*n* = 1} (90); low serum magnesium and low-grade inflammation—high-sensitive C-reactive protein {*n* = 1} (108); low serum potassium {*n* = 1}; blood homocysteine {*n* = 1} (102)	Sickle cell {*n* = 1} (72); Alpha thalassemia {*n* = 1} (71)
Endemic infections {*n* = 3}	HIV infection—lower SBP {*n* = 1} (84); Childhood Malaria infection—lower SBP {*n* = 1} (47); Whipworm infestation—higher SBP {*n* = 1} (87)	

#### Traditional risk factors

Sex (*n* = 53), age (*n* = 45), body mass index (*n* = 45), and other anthropometric measures (*n* = 15) were the most commonly studied traditional risk factors for HTN. Other important traditional risk factors include alcohol/tobacco use (*n* = 8), diet (*n* = 9), level of physical activity (*n* = 7), genetic (*n* = 7), and cardiometabolic risk factors (*n* = 7)—Table 3.

***Sex:*** Of 53 studies, nearly half 26 (49%) showed a significant association with HTN. Of those 26 studies, a slight majority 16 (62%) showed that females had a higher risk of HTN than males, and obesity and an unhealthy diet were the major factors to account for the gender differences.

***Age:*** Of 45 studies, a majority 32 (71%) showed a significant association with HTN, and 97% of these 32 studies showed that older age was associated with HTN.

***Body Mass Index (BMI) and other anthropometric measures:*** Of 59 studies, a majority 49 (83%) had a significant association with HTN. However, 10 (17%) studies did not find a significant association with HTN. These 10 studies were all cross-sectional, school-based, and the majority were conducted in urban settings.

***Diet:*** All 9 studies reporting on various dietary factors found significant associations with HTN. The studies assessed a deficiency of macronutrients, micronutrients, and minerals; excessive consumption of sugar, salt, and fats; and low consumption of vegetables and fruits.

***Physical inactivity:*** Of 7 studies, 4 (71%) found a significant association between physical inactivity and HTN. However, 1 study (14%) surprisingly showed that physical inactivity was associated with normal BP.

***Cardiometabolic risk factors:*** All 7 studies reporting various cardiometabolic risks found significant associations with HTN. The identified cardiometabolic risk factors were lower eGFR, higher pulse wave velocity, high fasting glucose, high resting pulse, and higher blood homocysteine, and these risk factors tended to cluster in females.

***Heredity:*** Of 8 studies, 6 (75%) showed a significant association with HTN. However, the majority 5 (83%) relied on self-reported family history of HTN. Importantly, one study using human genome data from the *Birth to Twenty cohort* (South Africa) showed that the NOSIAP, MYRE, and POCIB genes were associated with HTN in South African adolescents.

#### Non-traditional risk factors

These are factors related to socio-economic environmental and endemic diseases which are related to HTN.

Socioeconomic status and environmental factors (*n* = 21) as well as maternal and natal factors (*n* = 15) were the most commonly reported non-traditional risk factors. Other important non-traditional risks were chronic inflammation, blood composition, diseases of the blood (*n* = 5), and endemic infections (*n* = 3) (Table 3).

***Socioeconomic and environmental factors:*** Of 21 studies, 17 (81%) found a significant association between socioeconomic and environmental factors with HTN. Of the 17 studies, 12 (71%) found higher socioeconomic status and 5 (29%) found lower socioeconomic as a significant risk for HTN.

***Maternal and natal factors:*** Of 15 studies, majority 12 (80%) found a significant association between maternal and natal factors with HTN. Of the 12 studies, 5 (42%) assessed maternal factors, including events before or during pregnancy, including maternal exposure to mercury, maternal HTN, and malaria in pregnancy. Seven studies (58%) assessed the association between events occurring during or after birth, including low birth weight, weight gain after birth and during early childhood, and higher BP at a young age.

***Blood disorders:*** Of 5 studies, 3 (60%) found a significant association with HTN. Of the 3 studies, the reported risks were higher hemoglobin concentration (90), lower serum magnesium (108), lower serum potassium, and higher C-reactive protein levels (102, 109).

***Endemic infections:*** Of the 3 studies on HIV, malaria and whipworm infestation, only whipworm infestation was significantly associated with HTN (85).

#### Complications of hypertension

Only 3 studies assessed potential complications of HTN among adolescents in SSA. Two studies found no significant association between HTN and cognitive decline (57), proteinurias, left ventricular hypertrophy, and retinopathy (39). One study found a significant association between HTN and microalbuminuria (54) (Table 4).

**Table 4:** Summary of studies on complication of hypertension among adolescents aged 10–19 years in sub-Saharan Africa.

Author	Year	Sample size	Prevalence of complication (end organ damage)	Association with hypertension
Lyngdoh (57)	2013	407	Cognitive decline using multiple cognitive tests	Not significant
Mijinyawa (39)	2008	968	Proteinuria (30 mg/dl)—present in 15 (21.4%) out of 70 with high BP.	Not significant
			Left ventricular hypertrophy—present in 0 (0%) out of 41 with high BP and available for ECG.	
			Retinopathy—present in 0 (0%) out of 70 with high BP and available for fundoscopy.	
Okpere (54)	2012	615	Microalbuminuria—present in 12 (70.6%) out of 17 with hypertension	Significant (*p* = 0.001)

## Discussion

In this systematic review, we aimed to determine the burden, risk factors, and complications of HTN among adolescents in SSA.

The prevalence of HTN among adolescents in SSA was found to be high with varying estimates across studies. Overall, prevalence estimates from recently published studies were higher than those published in the past. This finding persisted even after taking into account of other possible reasons including differences in the devices used to measure BP and guidelines for defining HTN. In addition, studies with significantly higher prevalence estimates came from Southern Africa region, and overweight/obesity among girls was the major reported risk factor (62, 83, 88, 103, 104). Similar findings were observed in the previous systematic reviews (17, 18). The finding suggests that the burden of HTN is increasing and could explain the rising burden of CVD among young adults (17, 18, 112–114).

Nearly 90% of the included studies had their BP measurements obtained in one day contrary to the standard recommendations which require taking multiple BP measurements on at least 2 separate occasions (days) (11, 110). This raises concern about the accuracy of the HTN prevalence estimates and a potential possibility of an overestimated HTN burden (3). Adolescents are more likely than adults to experience white coat HTN (4, 115, 116). Moreover, majority of adolescents in SSA have never had their BP measured and are therefore more prone to the “white coat effect”, particularly when the BP measurements are taken on one occasion (4). This observation underscores the importance of following the standard BP measurement procedures particularly in this population to ensure accurate estimates.

Non-traditional risk factors related to poverty and tropical diseases may be important drivers of HTN among adolescents in SSA despite being under-reported and/or their role being less acknowledged (6, 15). We found relatively fewer studies that assessed/reported non-traditional risk factors, likely reflecting low awareness of their potential role on HTN in this population. In addition, the scarcity of long-term cohort studies among adolescents in SSA could partly explain this finding since most of the non-traditional risks involve tracking long term exposures occurring in one's lifetime (5, 15, 85, 87). However, it is worth noting that majority of studies assessing non-traditional risk factors often reported significant associations with HTN. This observation underscores the importance of the non-traditional risk factors although we cannot rule out publication bias.

The majority of the included studies were conducted in school settings among apparently healthy adolescents. This finding may reflect the willingness of schools and/or students to participate in research and health related interventions. It highlights the importance of schools as a potentially suitable platform for CVD prevention interventions targeting adolescents (117). Such interventions could be integrated into existing school health programs, and address multiple aspects of cardiovascular health including a healthy diet, physical exercise, and body weight control (117). BP measurement in schools might also help to raise awareness of the relevance of cardiovascular health for adolescents among teachers, parents, and entire communities. In addition, this finding points to the scarcity of hospital-based studies among adolescents in SSA where the epidemiology of HTN could be different.

Only three studies assessed/reported potential complications of HTN among adolescents in SSA (39, 54, 57), and mostly they found little or no complications. This may be due to using less-sensitive tools for the detection of HTN-related complications. For instance, in one of the three studies, left ventricular hypertrophy was assessed by electrocardiography which is known to have a sensitivity of less than 35% (39, 118). Tools with higher sensitivity and specificity to increase our awareness and knowledge of complications of HTN will help to determine the public health importance of HTN among adolescents in SSA.

Our study should be viewed in the context of the following strengths and limitations. A strength of our study is that we searched from multiple databases since their inception (from 1946). The wider review time frame allowed us to retrieve more studies published at widely ranging time points, hence giving a wider scope on how HTN estimates and their determinants have evolved over time. Our review focused on studies involving adolescents, and our findings provide useful information specific to this population. This is the first review to look at potential role of non-traditional risk factors and complications of hypertension among adolescents in SSA.

Our study has limitations. First, the retrieved studies came from only 14 (29%) of all 48 countries in SSA. The majority of studies came from Nigeria and South Africa, which are the two most advanced economies in SSA. Further, there were significant differences in prevalence estimates by African regions from which the studies came from. In this regard, our findings may not be generalizable to all countries in SSA. Secondly, a majority of studies were cross-sectional in design and therefore it is difficult to determine the temporal association with the reported risk factors. Thirdly, we cannot rule out publication bias, particularly for studies assessing/reporting non-traditional risk factors. And lastly, we did not conduct meta-analysis due to heterogeneity in study methods and settings/geography among the included studies.

### Added value and implications of these findings

To our understanding, this is the second systematic review including adolescent only data in SSA region (16). Our review is updating the previous three reviews and has mapped available literature in SSA and highlighted existing knowledge/data gaps (16–18). These findings underscore the importance of accurate BP measurement and diagnosis of HTN in adolescents, both in research and clinical settings. Since HTN during adolescence is associated with the growing HTN/CVD epidemic among adults, broadly these findings will help us to design a CVD prevention intervention targeting adolescents in SSA, particularly in school settings.

## Conclusion

HTN among adolescents in SSA is high, and non-traditional risk factors may be an important driver. Longitudinal observational and interventional studies are needed to clearly define the causes and complications of HTN in adolescents in SSA. In addition, governments and healthcare systems should provide the resources and accountability necessary for regular and proper BP measurements for adolescents in health facilities.

## Data Availability

The raw data supporting the conclusions of this article will be made available by the authors, without undue reservation.

## Supplementary material

SUPPLEMENTARY DATA SHEET 1

Search methods for rates, risk factors, and consequences of high blood pressure among adolescents in sub-Saharan Africa.

SUPPLEMENTARY DATA SHEET 2

List of publications whose full texts were not immediately available for review.

SUPPLEMENTARY DATA SHEET 3

Summary table of hypertension guidelines used in published studies among adolescents aged 10–19 years in sub-Saharan Africa.

## References

[1] Song P Zhang Y Yu J Zha M Zhu Y Rahimi K Global prevalence of hypertension in children: a systematic review and meta-analysis. JAMA Pediatr. (2019) 173(12):1154–63. 10.1001/jamapediatrics.2019.3310 31589252 PMC6784751

[2] UNICEF. The state of World’s Children—Investing in adolescents for breaking the cycles of poverty and inequity (2011). Available at: https://www.unicef.org/reports/state-worlds-children-2011

[3] Falkner B. Hypertension in children and adolescents: epidemiology and natural history. Pediatr Nephrol. (2010) 25:1219–24. 10.1007/s00467-009-1200-3 19421783 PMC2874036

[4] Nsanya MK Ayieko P Hashim R Mgema E Fitzgerald D Kapiga S Sustained high blood pressure and 24-h ambulatory blood pressure monitoring in Tanzanian adolescents. Sci Rep. (2021) 11:8397. 10.1038/s41598-021-87996-0 33864003 PMC8052360

[5] Alissa EM Ferns GA. Heavy metal poisoning and cardiovascular disease. J Toxicol. (2011) 2011:870125–46. 10.1155/2011/870125 21912545 PMC3168898

[6] Bedu-Addo G Alicke M Boakye-Appiah JK Abdul-Jalil I van der Giet M Schulze MB In utero exposure to malaria is associated with metabolic traits in adolescence: the agogo 2000 birth cohort study. J Infect. (2017) 75(5):455–63. 10.1016/j.jinf.2017.08.010 28851533

[7] Brook RD Weder AB Rajagopalan S. “Environmental hypertensionology” the effects of environmental factors on blood pressure in clinical practice and research. J Clin Hypertens. (2011) 13(11):836–42. 10.1111/j.1751-7176.2011.00543.x PMC8108751 22051429

[8] Mandy M Nyirenda M. Developmental origins of health and disease: the relevance to developing nations. Int Health. (2018) 10(2):66–70. 10.1093/inthealth/ihy006 29528398 PMC5856182

[9] Rajagopalan S Landrigan PJ. Pollution and the heart. N Engl J Med. (2021) 385(20):1881–92. 10.1056/nejmra2030281 34758254

[10] Fishman B Grossman E Zucker I Orr O Lutski M Bardugo A Adolescent hypertension and risk for early-onset type 2 diabetes: a nationwide study of 1.9 million Israeli adolescents. Diabetes Care. (2020) 44(1):e6–8. 10.2337/dc20-1752 33148634

[11] Flynn JT Falkner BE. New clinical practice guideline for the management of high blood pressure in children and adolescents. Hypertension. (2017) 70(4):683–6. 10.1161/HYPERTENSIONAHA.117.10050 28827475

[12] Leiba A Fishman B Twig G Gilad D Derazne E Shamiss A Association of adolescent hypertension with future end-stage renal disease. JAMA Intern Med. (2019) 179(4):517–23. 10.1001/jamainternmed.2018.7632 30801616 PMC6450304

[13] Urbina EM Mendizábal B Becker RC Daniels SR Falkner BE Hamdani G Association of blood pressure level with left ventricular mass in adolescents SHIP AHOY. Hypertension. (2019) 74(3):590–6. 10.1161/HYPERTENSIONAHA.119.13027 31327264

[14] Kagura J Adair LS Musa MG Pettifor JM Norris SA. Blood pressure tracking in urban black South African children: birth to twenty cohort. BMC Pediatr. (2015) 15(1):78–85. 10.1186/s12887-015-0402-z 26173634 PMC4502542

[15] Naidoo S Kagura J Fabian J Norris SA Commentary SE. Early life factors and longitudinal blood pressure trajectories are associated with elevated blood pressure in early adulthood. Hypertension. (2019) 73(2):301–9. 10.1161/HYPERTENSIONAHA.118.11992 30580689

[16] Chen A Waite L Mocumbi AO Chan YK Beilby J Ojji DB Elevated blood pressure among adolescents in sub-Saharan Africa: a systematic review and meta-analysis. Lancet Glob Health. (2023) 11(8):e1238–48. 10.1016/S2214-109X(23)00218-8 37474231

[17] Crouch SH Soepnel LM Kolkenbeck-Ruh A Maposa I Naidoo S Davies J Paediatric hypertension in Africa: a systematic review and meta-analysis. EClinicalMedicine. (2022) 43:101229. 10.1016/j.eclinm.2021.101229 34917909 PMC8665406

[18] Noubiap JJ Essouma M Bigna JJ Jingi AM Aminde LN Nansseu JR. Prevalence of elevated blood pressure in children and adolescents in Africa: a systematic review and meta-analysis. Lancet Public Health. (2017) 2(8):e375–86. 10.1016/S2468-2667(17)30123-8 29253478

[19] Bramer WM Rethlefsen ML Kleijnen J Franco OH. Optimal database combinations for literature searches in systematic reviews: a prospective exploratory study. Syst Rev. (2017) 6(1):1–12. 10.1186/s13643-017-0644-y 29208034 PMC5718002

[20] Grossetta Nardini HK Wang L. The Yale MeSH Analyzer (2018). http://mesh.med.yale.edu/

[21] Wells G Shea B O’Connell D Peterson J Welch V Losos M Newcastle-Ottawa Quality Assesment Scale Cohort Studies (2021). Available at: https://www.ohri.ca/programs/clinical_epidemiology/oxford.asp

[22] Page MJ McKenzie JE Bossuyt PM Boutron I Hoffmann TC Mulrow CD The PRISMA 2020 statement: an updated guideline for reporting systematic reviews. BMJ. (2021) 372:n71. 10.1136/bmj.n71 33782057 PMC8005924

[23] Akinkube OO Ojo AO. The systemic blood pressure in a rural Nigerian population. Trop Geogr Med. (1968) 20:347–56. 5707221

[24] Johnson TO. Arterial blood pressures and hypertension in an urban African population sample. Br J Prev Soc Med. (1971) 25(1):26–33. 10.1136/jech.25.1.26 5551230 PMC478626

[25] Akinkugbe A. Arterial pressures in non-pregnant women of child-bearing age in Ile-Ife, Nigeria. Br J Obstet Gynaecol. (1976) 83:545–9. 10.1111/j.1471-0528.1976.tb00883.x 952782

[26] Ayobanji Ayoola E. Prevalence of adolescent hypertension in Nigeria. Niger J Paediatr. (1979) 6(1):18–26.

[27] Abu-Bakare A Oyaide SM. Blood pressure levels in Nigerian school girls. J Trop Pediatr. (1983) 29:225–9. 10.1093/tropej/29.4.225 6632045

[28] Idahosa PE. Blood pressure pattern in urban edos. J Hypertens. (1985) 3(Suppl. 3):S379–81. PMID: . 2856744

[29] Eferakeya AE. Arterial blood pressures in Benin city (Nigeria) children. Public Health. (1986) 100(3):174–9. 10.1016/S0033-3506(86)80031-2 3737865

[30] Adams-Campbell LL Ukoli F Young MP Omene J Nwankwo M Haile GT An epidemiological assesment of blood pressure determinants in an adolescent population of Nigerians. J Hypertens. (1987) 5:575–80. 10.1097/00004872-198710000-00011 3429862

[31] Kitange HM Swai ABM Masuki G Kilima PM Alberti KGMM McLarty DG. Coronary heart disease risk factors in sub-Saharan Africa: studies in Tanzanian adolescents. J Epidemiol Community Health. (1993) 47(4):303–7. 10.1136/jech.47.4.303 8228767 PMC1059798

[32] Muraguri PW McLigeyo SO Kayima JK. Proteinuria, other slected urinary abnormalities and hypertension among teenage secondary school students in Nairobi, Kenya. East Afr Med J. (1997) 74(8):467–73. PMID: . 9487409

[33] Kane A Diof N. Arterial pressure and body mass index of children and adolescents in a rural area of Thiadiaye, Senegal. Dakar Med. (1998) 43:83–9. PMID: . 9827163

[34] Longo-Mbenza B Ngiyulu R Bayekula M Vita EK Nkiabungu FB Seghers KV Low birth weight and risk of hypertension in African school children. J Cardiovasc Risk. (1999) 6(5):311–4. 10.1177/204748739900600507 10534134

[35] Schutte AE Van Rooyen JM Huisman HW Kruger HS Malan NT De Ridder JH. Dietary risk markers that contribute to the aetiology of hypertension in black South African children: the THUSA BANA study. J Hum Hypertens. (2003) 17(1):29–35. 10.1038/sj.jhh.1001508 12571614

[36] Schutte AE Van Rooyen JM Huisman HW Kruger HS De Ridder JH. Factor analysis of possible risks for hypertension in a black South African population. J Hum Hypertens. (2003) 17(5):339–48. 10.1038/sj.jhh.1001553 12756407

[37] Van Rooyen JM Kruger HS Huisman HW Schutte AE Malan NT Schutte R. Early cardiovascular changes in 10- to 15-year-old stunted children: the transition and health during urbanization in South Africa in children study. Nutrition. (2005) 21(7–8):808–14. 10.1016/j.nut.2004.12.007 15975488

[38] Chiolero A Madeleine G Gabriel A Burnier M Paccaud F Bovet P. Prevalence of elevated blood pressure and association with overweight in children of a rapidly developing country. J Hum Hypertens. (2007) 21(2):120–7. 10.1038/sj.jhh.1002125 17136104

[39] Mijinyawa MS Iliyasu Z Borodo MM. Prevalence of hypertension among teenage students in Kano, Nigeria. Niger J Med. (2008) 17(2):173–8. 10.4314/njm.v17i2.37378 18686834

[40] Monyeki KD Kemper HCG Makgae PJ. Relationship between fat patterns, physical fitness and blood pressure of rural South African children: ellisras longitudinal growth and health study. J Hum Hypertens. (2008) 22(5):311–9. 10.1038/jhh.2008.3 18273041

[41] Cournil A Coly AN Diallo A Simondon KB. Enhanced post-natal growth is associated with elevated blood pressure in young Senegalese adults. Int J Epidemiol. (2009) 38(5):1401–10. 10.1093/ije/dyp255 19661279

[42] Mijinyawa MS Abdu A Habib A. Pattern of blood pressure in adolescents. Sahel Med J. (2009) 12(4):159–64. 10.4314/smj2.v12i4.55694

[43] Odey F Anah M Ansa V Ogbeche J Meremikwu M Ekanem E. Pre-hypertension and hypertension in apparently healthy adolescents in Calabar, Nigeria. Global J Commun Med. (2009) 2(1–2):13–20. 10.4314/gjcm.v2i1-2.47924

[44] Ansa V Anah M Odey F Mbu P Agbor E. Relationship between parental socio-economic Status and casual blood pressure in coastal Nigerian adolescents. West Afr J Med. (2011) 29(3):146–52. 10.4314/wajm.v29i3.68211 20665456

[45] Ejike CECC Ugwu CE Ezeanyika LUS. Variations in the prevalence of point (pre)hypertension in a Nigerian school-going adolescent population living in a semi-urban and an urban area. BMC Pediatr. (2010) 10:13. 10.1186/1471-2431-10-13 20214768 PMC2841152

[46] Kruger HS Pretorius R Schutte AE. Stunting, adiposity, and low-grade inflammation in African adolescents from a township high school. Nutrition. (2010) 26(1):90–9. 10.1016/j.nut.2009.10.004 20005466

[47] Chiolero A Paradis G Madeleine G Hanley JA Paccaud F Bovet P. Birth weight, weight change, and blood pressure during childhood and adolescence: a school-based multiple cohort study. J Hypertens. (2011) 29(10):1871–9. 10.1097/HJH.0b013e32834ae396 21881523

[48] Hawkesworth S Walker CG Sawo Y Fulford AJC Jarjou LMA Goldberg GR Nutritional supplementation during pregnancy and offspring cardiovascular disease risk in the Gambia. Am J Clin Nutr. (2011) 94(6):1853. 10.3945/ajcn.110.000877 21677054

[49] Mamabolo R Van Rooyen J Schutte A Monyeki M Kruger H. Association between blood pressure, measures of body composition and lifestyle factors in township adolescents, North-West Province, South Africa. Afr J Phys Health Educ Recreat Dance. (2011) 17(1):51–68. 10.4314/ajpherd.v17i1.65245

[50] Meehan KA Bankoski AJ Tejan E Ansumana R Bangura U Stenger DA Hypertension in Bo, Sierra Leone. Ethn Dis. (2011) 21(2):237–42. PMID: . 21749030

[51] Bukabau JB Makulo JRR Pakasa NM Cohen EP Lepira FB Kayembe PK Chronic kidney disease among high school students of Kinshasa. BMC Nephrol. (2012) 13:24–9. 10.1186/1471-2369-13-24 22559052 PMC3464656

[52] Griffiths PL Sheppard ZA Johnson W Cameron N Pettifor JM Norris SA. Associations between household and neighbourhood socioeconomic status and systolic blood pressure among urban South African adolescents. J Biosoc Sci. (2012) 44(4):433–58. 10.1017/S0021932012000107 22490826

[53] Okoh BA Alikor EA Akani N. Prevalence of hypertension in primary schoolchildren in Port Harcourt, Nigeria. Paediatr Int Child Health. (2012) 32(4):208–12. 10.1179/2046905512Y.0000000039 23164295

[54] Okpere AN Anochie IC Eke FU. Prevalence of microalbuminuria among secondary school children. Afr Health Sci. (2012) 12(2):140–7. 10.4314/ahs.v12i2.10 23056019 PMC3462545

[55] Oyewole OO Oritogun KS. Pre-hypertension and hypertension in adolescence: how much does it occur in a Nigerian community? West Afr J Med. (2012) 31(2):71–5. http://www.ncbi.nlm.nih.gov/pubmed/23208473 23208473

[56] Goon D Amusa L Mhlongo D Khoza L Any-Anwu F. Elevated blood pressure among rural South African children in Thohoyandou, South Africa. Iran J Public Health. (2013) 42(5):489–96. http://ijph.tums.ac.ir 23802106 PMC3684457

[57] Lyngdoh T Viswanathan B Kobrosly R Van Wijngaarden E Huber B Davidson PW Blood pressure and cognitive function: a prospective analysis among adolescents in Seychelles. J Hypertens. (2013) 31(6):1175–82. 10.1097/HJH.0b013e3283604176 23572201 PMC3874141

[58] Okpere AN Anochie IC Eke FU. Pattern of blood pressure and hypertension in adolescents in Port Harcourt, Nigeria. West Afr J Med. (2013) 32(2):93–8. PMID: . 23913495

[59] Ujunwa FA Ikefuna AN Nwokocha AR Chinawa JM. Hypertension and prehypertension among adolescents in secondary schools in Enugu, South East Nigeria. Ital J Pediatr. (2013) 39:1–6. 10.1186/1824-7288-39-70 24180427 PMC4228429

[60] Mushengezi B Chillo P. Association between body fat composition and blood pressure level among secondary school adolescents in Dar es Salaam, Tanzania. Pan Afr Med J. (2014) 19:327–38. 10.11604/pamj.2014.19.327.5222 25918567 PMC4405073

[61] Okagua J Akani N. Prevalence of hypertension in school going adolescents in rural areas of Rivers State, South-South Nigeria. Niger Health J. (2014) 14(4):157–64.

[62] Nkeh-Chungag B Sekokotla AM. Prevalence of hypertension in 13–17 years old in MTHATHA South Africa. Cent Eur J Public Health. (2015) 23(1):59–64. 10.21101/cejph.a3922 26036100

[63] Odunaiya NA Louw QA Grimmer KA. Are lifestyle cardiovascular disease risk factors associated with pre-hypertension in 15–18 years rural Nigerian youth? A cross sectional study. BMC Cardiovasc Disord. (2015) 15(1):144–54. 10.1186/s12872-015-0134-x 26537355 PMC4632346

[64] Oyeyemi AY Usman MA Oyeyemi AL Jaiyeola OA. Casual blood pressure of adolescents attending public secondary schools in Maiduguri, Nigeria. Clin Hypertens. (2015) 21(3):16–21. 10.1186/s40885-015-0026-5 26893926 PMC4750820

[65] Ratovoson R Rasetarinera OR Andrianantenaina I Rogier C Piola P Pacaud P. Hypertension, a neglected disease in rural and urban areas in Moramanga, Madagascar. PLoS One. (2015) 10:9. 10.1371/journal.pone.0137408 PMC4565657 26355997

[66] Awotidebe A Monyeki MA Moss SJ Strydom GL Amstrong M Kemper HCG. Relationship of adiposity and cardiorespiratory fitness with resting blood pressure of South African adolescents: the PAHL study. J Hum Hypertens. (2016) 30(4):245–51. 10.1038/jhh.2015.81 26202691

[67] Munthali RJ Kagura J Lombard Z Norris SA. Childhood adiposity trajectories are associated with late adolescent blood pressure: birth to twenty cohort. BMC Public Health. (2016) 16(1):665–75. 10.1186/s12889-016-3337-x 27473865 PMC4966706

[68] Strassman BI Smith CS Vincenz C. Does puberty influence systolic blood pressure independent of the effects of adolescent growth and body size? Am J Phys Anthropol. (2016) 159:305. 10.1002/ajpa.22955

[69] Uwaezuoke S Okoli C Ubesie A Ikefuna A. Primary hypertension among a population of Nigerian secondary school adolescents: prevalence and correlation with anthropometric indices: a cross-sectional study. Niger J Clin Pract. (2016) 19(5):649–54. 10.4103/1119-3077.188706 27538555

[70] Alicke M Boakye-Appiah JK Abdul-Jalil I Henze A Van Der Giet M Schulze MB Adolescent health in rural Ghana: a cross-sectional study on the co-occurrence of infectious diseases, malnutrition and cardio-metabolic risk factors. PLoS One. (2017) 12(7):e0180436–51. 10.1371/journal.pone.0180436 28727775 PMC5519039

[71] Etyang AO Khayeka-Wandabwa C Kapesa S Muthumbi E Odipo E Wamukoya M Blood pressure and arterial stiffness in Kenyan adolescents with *α*+thalassemia. J Am Heart Assoc. (2017) 6(4):e005613–20. 10.1161/JAHA.117.005613 28381468 PMC5533038

[72] Etyang AO Wandabwa CK Kapesa S Muthumbi E Odipo E Wamukoya M Blood pressure and arterial stiffness in Kenyan adolescents with the sickle cell trait. Am J Epidemiol. (2018) 187(2):199–205. 10.1093/aje/kwx232 28992220 PMC5860135

[73] Ezeudu CE Chukwuka JO Ebenebe JC Igwe WC Egbuonu I. Hypertension and prehypertension among adolescents attending secondary schools in urban area of South-east, Nigeria. Pan Afr Med J. (2018) 31:145–54. 10.11604/pamj.2018.31.145.15994 31037205 PMC6462383

[74] Hendry LM Sahibdeen V Choudhury A Norris SA Ramsay M Lombard Z. Insights into the genetics of blood pressure in black South African individuals: the birth to twenty cohort. BMC Med Genom. (2018) 11:2. 10.1186/s12920-018-0321-6 PMC5773038 29343252

[75] Isezuo KO Jiya NM Audu LI Ibitoye PK Sani UM Yusuf T Blood pressure pattern and the relationship with body mass index among apparently healthy secondary—school students in Sokoto Metropolis, Nigeria. SAJCH S Afr J Child Health. (2018) 12(3):105–10. 10.7196/SAJCH.2018.v12i3.1475

[76] Leyvraz M Wahlen R Bloetzer C Paradis G Bovet P Chiolero A. Persistence of elevated blood pressure during childhood and adolescence: a school-based multiple cohorts study. J Hypertens. (2018) 36(6):1306–10. 10.1097/HJH.0000000000001699 29517559

[77] Masocha V Czyż SH Moss SJ Monyeki AM. Two-year changes in body composition, physical activity and selected metabolic risk factors among adolescents living in tlokwe municipality area, north west province, South Africa: the PAHL study. S Afr J Res Sport Phys Educ Recreation. (2018) 40(2):99–114. 10.1123/jpah.2018-0535

[78] Nakiriba R Mayega RW Piloya T Nabukeera-Barungi N Idro R. Prevalence and factors associated with dysglycemia among girls in selected boarding secondary schools in Wakiso district, Uganda. Adolesc Health Med Ther. (2018) 9:167–76. 10.2147/ahmt.s178746 30464672 PMC6211585

[79] Omisore AG Omisore B Abioye-Kuteyi EA Bello IS Olowookere SA. In-school adolescents’ weight status and blood pressure profile in south-western Nigeria: urban-rural comparison. BMC Obes. (2018) 5:2 (2018). 10.1186/s40608-018-0179-3 PMC5787246 29423239

[80] Abu OO Raji YR Amodu OK. Risk factors for chronic kidney disease among in-school adolescents in Ibadan, Southwest, Nigeria. Sahel Med J. (2019) 22(2):64. 10.4103/smj.smj_21_18

[81] Adeomi AA Adelusi IO Adedeji PO Awofeso AE Oroleye OO Gbadegesin DL. Nutritional status and cardiometabolic health among adolescents; findings from southwestern Nigeria. BMC Nutr. (2019) 5:45. 10.1186/s40795-019-0308-5 32153958 PMC7050742

[82] Amponsem-Boateng C Zhang W Oppong TB Opolot G Kyere EKD. A cross-sectional study of risk factors and hypertension among adolescent senior high school students. Diabetes Metab Syndr Obes Targets Ther. (2019) 12:1173–80. 10.2147/DMSO.S213552 PMC6662518 31413610

[83] Chungag A Tata CM Sewani-Rusike CR Nel W Nkeh-Chungag BN. Ellisras longitudinal study 2017: association of hypertension with increasing levels of adiposity in 10- to 14-year-old boys and girls in the eastern cape (ELS 31). Cardiovasc J Afr. (2019) 30(5):258–61. 10.5830/CVJA-2019-017 31746941 PMC8802374

[84] Frigati L Mahtab S Nourse P Ray P Perrazzo S Machemedze T Prevalence of risk factors for chronic kidney disease in South African youth with perinatally acquired HIV. Pediatr Nephrol. (2019) 34(2):313–8. 10.1007/s00467-018-4080-6 30219929 PMC6529608

[85] Lule SA Namara B Akurut H Lubyayi L Nampijja M Akello F Blood pressure risk factors in early adolescents: results from a Ugandan birth cohort. J Hum Hypertens. (2019) 33(9):679–92. 10.1038/s41371-019-0178-y 30804461 PMC6760975

[86] Lule SA Mentzer AJ Namara B Muwenzi AG Nassanga B Kizito D A genome-wide association and replication study of blood pressure in Ugandan early adolescents. Mol Genet Genomic Med. (2019) 7(10):e950. 10.1002/mgg3.950 PMC6785527 31469255

[87] Lule SA Namara B Akurut H Muhangi L Lubyayi L Nampijja M Are birthweight and postnatal weight gain in childhood associated with blood pressure in early adolescence? Results from a Ugandan birth cohort. Int J Epidemiol. (2019) 48(1):148–56. 10.1093/ije/dyy118 29982658 PMC6380421

[88] Nkwana MR Monyeki KD Monyeki SM Makata TT Monyeki JM. Ellisras lingitudinal study 2017: the association of fat patterning with blood pressure in polokwane private school children aged five to 15 years (ELS 22). Cardiovasc J Afr. (2019) 30(3):142–5. 10.5830/CVJA-2018-058 31139814

[89] Nsanya MK Kavishe BB Katende D Mosha N Hansen C Nsubuga RN Prevalence of high blood pressure and associated factors among adolescents and young people in Tanzania and Uganda. J Clin Hypertens. (2019) 21(4):470–8. 10.1111/jch.13502 PMC8030556 30811099

[90] Abiodun O Ladele A Olu-Abiodun O Ashipa T. Hypertension among adolescents in Nigeria: a retrospective study of adolescent university freshmen. Int J Adolesc Med Health. (2021) 33(5):20180287. 10.1515/ijamh-2018-0287 30875324

[91] Azupogo F Abizari AR Aurino E Gelli A Osendarp SJM Bras H Malnutrition, hypertension risk, and correlates: an analysis of the 2014 Ghana demographic and health survey data for 15–19 years adolescent boys and girls. Nutrients. (2020) 12(9):1–23. 10.3390/nu12092737 PMC7551149 32911770

[92] Katamba G Agaba DC Migisha R Namaganda A Namayanja R Turyakira E. Prevalence of hypertension in relation to anthropometric indices among secondary adolescents in Mbarara, Southwestern Uganda. Ital J Pediatr. (2020) 46(1):76–83. 10.1186/s13052-020-00841-4 32487198 PMC7268267

[93] Masocha V Monyeki MA Czyż SH. Longitudinal relationships between changes in body composition and changes in selected metabolic risk factors (abdominal obesity and blood pressure) among South African adolescents. PeerJ. (2020) 2020(6):e9331. 10.7717/peerj.9331 PMC7319020 32612883

[94] Mokgwathi M Mwita JC. Prevalence of hypertension and selected cardiovascular risk factors among adolescents in selected rural and urban secondary schools in Botswana. S Afr J Diabetes Vasc Dis. (2020) 7(1):23–28. 10.10520/EJC-1edab1a94e PMC8762763 31781715

[95] Raphadu TT Van Staden M Dibakwane WM Monyeki KD. A non-invasive investigation into the prevalence of higher than normal blood pressure, hypertension and the association between blood pressure and body weight in male and female adolescents in the Polokwane Local Municipality, Limpopo-South Africa: a cros. Children. (2020) 7(3):18. 10.3390/children7030018 32143272 PMC7140854

[96] Sungwa EE Kibona SE Dika HI Laisser RM Gemuhay HM Kabalimu TK Prevalence and factors that are associated with elevated blood pressure among primary school children in Mwanza Region, Tanzania. Pan Afr Med J. (2020) 37:283. 10.11604/pamj.2020.37.283.21119 33654510 PMC7896535

[97] Ugochukwu EF Onubogu CU Ofora VC Okeke KN Uju CM. Blood pressure profiles and determinants of hypertension among public secondary school students in Nnewi, Southeast Nigeria. Eur J Med Health Sci. (2020) 2:3. 10.24018/ejmed.2020.2.3.298

[98] Ukoh U Ujunwa F Muoneke U Manyike P Okike C Ibe B. Oscillometric blood pressure profile of adolescent secondary school students in Abakaliki metropolis. Ann Afr Med. (2020) 19(1):31–9. 10.4103/aam.aam_21_19 32174613 PMC7189881

[99] Akinbodewa AA Adejumo AO Lamidi OA Adeyemi O. Clustering of cardiometabolic risk factors among children and adolescents in a rural community in Ondo, Southwest Nigeria. J Trop Pediatr. (2021) 66(4):366–76. 10.1093/TROPEJ/FMZ075 31665517

[100] Ayogu RNB Nwodo CJ. Epidemiological characteristics of hypertension, impaired fasting capillary glucose and their comorbidity: a retrospective cross-sectional population-based study of rural adolescents in Southeast Nigeria. BMJ Open. (2021) 11(5):e041481. 10.1136/bmjopen-2020-041481 33952534 PMC8103371

[101] Chungag A Engwa GA Sewani-Rusike CR Nkeh-Chungag BN. Effect of seasonal variation on the relationship of indoor air particulate matter with measures of obesity and blood pressure in children. J Health Pollution. (2021) 11:30. 10.5696/2156-9614-11.30.210610 PMC8276733 34267997

[102] du Plessis JP Nienaber-Rousseau C Lammertyn L Schutte AE Pieters M Kruger HS. The relationship of circulating homocysteine with fibrinogen, blood pressure, and other cardiovascular measures in African adolescents. J Pediatr. (2021) 234:158–63.e2. 10.1016/j.jpeds.2021.03.034 33775664

[103] Engwa G Letswalo P Nkeh-Chungag B. Obesity, hypertriglycaemia and endothelial dysfunction are risk factors of hypertension in South African adolescents. J Hypertens. (2021) 39(March):2021. 10.1097/01.hjh.0000746524.27921.7e

[104] Letswalo BP Schmid-Zalaudek K Brix B Matjuda EN Klosz F Obernhumer N Cardiometabolic risk factors and early indicators of vascular dysfunction: a cross-sectional cohort study in South African adolescents. BMJ Open. (2021) 11(3):e042955. 10.1136/bmjopen-2020-042955 33737426 PMC7978086

[105] Lwabukuna WC Mgonda Y. Early clinical markers of metabolic syndrome among secondary school adolescents in Dar es Salaam, Tanzania. Tanzan J Health Res. (2021) 22(1):1–7. 10.4314/thrb.v22i1.3

[106] Meer R Boateng D Klipstein-Grobusch K Norris SA Kagura J. Incidence and correlates of high blood pressure from childhood to adulthood: the birth to twenty study. J Hypertens. (2022) 40(2):274–82. 10.1097/HJH.0000000000003004 34475345 PMC8728753

[107] Nganou-Gnindjio CN Essama DB Nkeck JR Tchebegna PY Tchatchouang KM Tankeu A Prevalence and factors associated with hypertension among school children and adolescents in urban and semi-urban areas in Cameroon. J Clin Hypertens. (2021) 23(8):1490–7. 10.1111/jch.14309 PMC8678760 34152698

[108] Sekokotla AM Iputo JE Sewani-Rusike CR Malema IM Adeniyi OV Goon DT Serum magnesium and high-sensitive c-reactive proteins in hypertensive, obese female school learners. West Indian Med J. (2021) 69(1):32–7. 10.7727/wimj.2015.292

[109] Shokunbi OS Ukangwa NA. Relationship of blood pressure status, dietary factors and serum electrolytes of in-school adolescents in Ilishan-Remo, Ogun State, Nigeria. Afr Health Sci. (2021) 21(4):1754–63. 10.4314/ahs.v21i4.32 35283970 PMC8889815

[110] NHLBI. The fourth report on the diagnosis, evaluation and treatment of high blood pressure in children and adolescents. Pediatrics. (2004) 114(2):555–76. 10.1542/peds.114.2.S2.555 15286277

[111] Thurston SW Bovet P Myers GJ Davidson PW Georger LA Shamlaye C Does prenatal methylmercury exposure from fish consumption affect blood pressure in childhood? NeuroToxicology. (2007) 28(5):924–30. 10.1016/j.neuro.2007.06.002 17659343 PMC2104472

[112] Kavishe B Biraro S Baisley K Vanobberghen F Kapiga S Munderi P High prevalence of hypertension and of risk factors for non-communicable diseases (NCDs): a population based cross-sectional survey of NCDS and HIV infection in northwestern Tanzania and Southern Uganda. BMC Med. (2015) 13:126–47. 10.1186/s12916-015-0357-9 26021319 PMC4476208

[113] Peck R Green E Mtabaji J Majinge C Smart L Downs J Hypertension related diseases as a common cause of hospital mortality in Tanzania. A 3 year prospective study. J Hypertens. (2014) 31(9):1806–11. 10.1097/HJH.0b013e328362bad7.HYPERTENSION-RELATED PMC4005815 23777761

[114] Peck R Baisley K Kavishe B Were J Mghamba J Smeeth L Decreased renal function and associated factors in cities, towns and rural areas of Tanzania: a community-based population survey. Trop Med Int Health. (2016) 21(3):393–404. 10.1111/tmi.12651 26644310 PMC4784164

[115] Myers MG McInnis NH Fodor GJ Leenen FHH. Comparison between an automated and manual sphygmomanometer in a population survey. Am J Hypertens. (2008) 21(3):280–3. 10.1038/ajh.2007.54 18219304

[116] Stergiou GS Boubouchairopoulou N Kollias A. Accuracy of automated blood pressure measurement in children evidence, issues, and perspectives. Hypertension. (2017) 69(6):1000–6. 10.1161/HYPERTENSIONAHA.116.08553 28438903

[117] Steinberger J Daniels SR Hagberg N Isasi RC Kellly AS Lloyd-Jones D Cardiovascular health promotion in children: challenges and opportunities for 2020 and beyond. Circulation. (2016) 134:e236–55. 10.1161/CIR.0000000000000441 27515136 PMC5218582

[118] Woroniecki RP Kahnauth A Panesar LE Supe-Markovina K. Left ventricular hypertrophy in pediatric hypertension: a mini review. Front Pediatr. (2017) 5(May):1–7. 10.3389/fped.2017.00101 28553631 PMC5425592

