# Supplementary material for: Hypertension among adolescents in sub-Saharan Africa: a systematic review
Source: Front Cardiovasc Med. 2023 Dec 7;10:1251817. doi: 10.3389/fcvm.2023.1251817 (PMC10754047; doi:10.3389/fcvm.2023.1251817)
Supplement: Supplementary file 1 [file Datasheet1.pdf]

Search methods for Rates, risk factors and consequences of high blood pressure among adolescents in sub-Saharan Africa

Correspondence: Andy Hickner, MSI, alh4014@med.cornell.edu.

This appendix reports the search methods for the review using the PRISMA extension for searching (*Rethlefsen ML, 2021*). This search narrative was last updated July 19, 2022.

Information Sources and Methods

MEDLINE ALL (Ovid), Embase (Ovid), Web of Science Core Collection (Editions = A&HCI, BKCI-SSH, BKCI-S, CCR-EXPANDED, ESCI, IC, CPCI-SSH, CPCI-S, SCI-EXPANDED, SSCI) (Clarivate), and African Index Medicus (Global Index Medicus) were searched on December 22, 2021. In addition, the first 200 results from Google Scholar were exported on December 22, 2021 using Harzing's Publish or Perish software. All databases were searched from inception. Results were imported into, and automatically deduplicated in, the Covidence systematic review software. (*Covidence systematic review software, Veritas Health Innovation, Melbourne, Australia, available at* www.covidence.org)

Search strategies

The strategy consists of three concepts, combined with Boolean operators: adolescents, sub-Saharan Africa, and high blood pressure. Both controlled vocabulary and free-text keywords were identified using the Yale MeSH Analyzer; proximity operators were incorporated in order to retrieve variations of phrases. No limits were used for any of the databases, (*Grossetta NHK, 2021*)

MEDLINE (Ovid)

(exp adolescent/ or (adolescen$ or teen$ or youth$).tw,kf.) and ((exp African Continental Ancestry Group/ not exp african americans/) or ((Africa$ or Angola$ or Benin$ or Botswan$ or Burkina Faso or Burundi$ or Cameroon$ or Canary Island$ or Cape Verde$ or Chad or Comoros or Congo$ or Djibouti$ or Equatorial Guinea$ or Eritrea$ or Ethiopia$ or Gabon$ or Gambia$ or Ghana$ or Guinea$ or Guinea Bissau or Ivory Coast or Cote d'Ivoire or Jamahiryia or Kenya$ or Lesotho or Liberia$ or Madagascar or malagasy or Malawi or Mali or Mauritania$ or Mauritius or Mozambique or Mocambique or Mozambique or Namibia$ or Niger$ or Principe or Reunion or Rwanda$ or Sao Tome or Senegal$ or Seychelles or Sierra Leone$ or Somalia$ or ((Saint or St or "St.") adj1 Helena) or Sudan$ or Swaziland or Tanzania$ or Togo or Uganda$ or Zaire$ or Zambia$ or Zimbabwe$ or sub-Saharan) not (african american$ or african-american$ or guinea pig$ or aspergillus niger)).tw,kf.) and (exp hypertension/ or (arterial hypertens$ or blood pressure$).tw,kf.)

Embase (Ovid)

(exp adolescent/ or (adolescen$ or teen$ or youth$).tw,kf.) and ((exp african/ not exp african american/) or ((Africa$ or Angola$ or Benin$ or Botswan$ or Burkina Faso or Burundi$ or Cameroon$ or Canary Island$ or Cape Verde$ or Chad or Comoros or Congo$ or Djibouti$ or Equatorial Guinea$ or Eritrea$ or Ethiopia$ or Gabon$ or Gambia$ or Ghana$ or Guinea$ or Guinea Bissau or Ivory Coast or Cote d'Ivoire or Jamahiryia or Kenya$ or Lesotho or Liberia$ or

Madagascar or malagasy or Malawi or Mali or Mauritania$ or Mauritius or Mozambique or Mocambique or Mozambique or Namibia$ or Niger$ or Principe or Reunion or Rwanda$ or Sao Tome or Senegal$ or Seychelles or Sierra Leone$ or Somalia$ or ((Saint or St or "St.") adj1 Helena) or Sudan$ or Swaziland or Tanzania$ or Togo or Uganda$ or Zaire$ or Zambia$ or Zimbabwe$ or sub-Saharan) not (african american$ or african-american$ or guinea pig$ or aspergillus niger)).tw,kf.) and (exp hypertension/ or (arterial hypertens$ or blood pressure$).tw,kf.)

Web of Science (A&HCI, BKCI-SSH, BKCI-S, CCR-EXPANDED, ESCI, IC, CPCI-SSH, CPCI-S, SCI-EXPANDED, SSCI) (Clarivate)

TS=((adolescen* or teen* or youth$) and ((Africa* or Angola* or Benin* or Botswan* or Burkina Faso or Burundi* or Cameroon* or (Canary NEAR/1 island*) or (Cape NEAR/2 Verde*) or Chad or Comoros or Congo* or Djibouti* or (Equatorial NEAR/1 Guinea) or Eritrea* or Ethiopia* or Gabon* or Gambia* or Ghana* or Guinea* or "Guinea Bissau" or "Ivory Coast" or "Cote d'Ivoire" or Jamahiriya or Kenya* or Lesotho or Liberia* or Madagascar or malagasy or Malawi or Mali or Mauritania* or Mauritius or Mozambique or Mocambique or Mozambique or Namibia* or Niger* or Principe or Reunion or Rwanda* or "Sao Tome" or Senegal* or Seychelles or "Sierra Leone" or Somalia* or "saint helena" or "St. Helena" or Sudan* or Swaziland or Tanzania* or Togo or Uganda* or Zaire* or Zambia* or Zimbabwe* or sub-Saharan) not (african-american or (african NEAR/1 american$) or (guinea NEAR/1 pig$) or "aspergillus niger")) and ((hypertens* or (blood NEAR/1 pressure*)) NOT (("portal hypertension" or "pulmonary hypertension") NOT "arterial hypertension")))

African Index Medicus (Global Index Medicus)

tw:((mh:("Adolescent") OR adolescen* OR teen* OR youth*) AND (mh:("hypertension") OR "arterial hypertension" OR "blood pressure"))

Other sources

Searched Google Scholar via Harzing's Publish or Perish, exported first 200 results

adolescents|teens africa|african -african-american -"african american" hypertension|"blood pressure"

Dates and times of export

All times US Eastern Standard

Databases, number of results, time/date of export

- African Index Medicus: 15, 9:12am on Dec 22, 2021
- Web of Science: 373, 9:30am on Dec 22, 2021
- MEDLINE ALL: 1882, 9:45am on Dec 22, 2021
- Embase: 1538, 9:58am on Dec 22, 2021

Total before deduplication: 3,808 (excludes Google Scholar*)

357 duplicates automatically removed by Covidence (excludes Google Scholar*)

Total to screen after deduplication: 3451 (excludes Google Scholar*)

Other sources

*Google Scholar: first 200, 8:52am on Dec 22, 2021

1 duplicate removed by Covidence

No limits were used.

Discussion of search methods

Ovid MEDLINE, Embase, and African Index Medicus were searched in order to identify relevant published literature. Embase and Web of Science Core Collection were searched in order to identify studies that had been presented as meeting abstracts but not yet published as a full-length journal article. Previous research has suggested this as the optimal minimum combination of databases for systematic reviews in the health sciences. Google Scholar was searched in order to identify potential grey literature (*Bramer WM, 2017*)

All sources were searched from dates of inception. The search was initially developed in MEDLINE, then translated to other databases using the Polyglot tool (*Clark JM, 2020*); the automatically translated searches were further adjusted by hand. Controlled vocabulary terms were exploded whenever possible.

Members of the study team identified a list of 12 MEDLINE-indexed studies during exploratory searching; these were used to test the strategies for MEDLINE, Embase, and Web of Science.

References

Bramer WM, Rethlefsen ML, Kleijnen J, Franco OH. Optimal database combinations for literature searches in systematic reviews: a prospective exploratory study. Syst Rev. 2017;6(1):245. Published 2017 Dec 6. doi:10.1186/s13643-017-0644-y

Clark JM, Sanders S, Carter M, Honeyman D, Cleo G, Auld Y, Booth D, Condron P, Dalais C, Bateup S, Linthwaite B, May N, Munn J, Ramsay L, Rickett K, Rutter C, Smith A, Sondergeld P, Wallin M, Jones M, Beller E. Improving the translation of search strategies using the Polyglot Search Translator: a randomized controlled trial. J Med Libr Assoc. 2020 Apr;108(2):195–207.

Grossetta Nardini HK, Wang L. The Yale MeSH Analyzer. 2021; http://mesh.med.yale.edu. Accessed October 28, 2021.

Rethlefsen ML, Kirtley S, Waffenschmidt S, Ayala AP, Moher D, Page MJ, Koffel JB; PRISMA-S Group. PRISMA-S: an extension to the PRISMA Statement for Reporting Literature Searches in Systematic Reviews. Syst Rev. 2021 Jan 26;10(1):39. doi: 10.1186/s13643-020-01542-z.
