# Supplementary material for: Hypertension among adolescents in sub-Saharan Africa: a systematic review
Source: Front Cardiovasc Med. 2023 Dec 7;10:1251817. doi: 10.3389/fcvm.2023.1251817 (PMC10754047; doi:10.3389/fcvm.2023.1251817)
Supplement: Supplementary file 2 [file Datasheet2.pdf]

List of 15 publications whose full texts were not available for review

1. Okagua, J.; Anochie, I. C. Blood pressure profile and hypertension in adolescents in Port Harcourt, Southern Nigeria. African Journal of Paediatric Nephrology // 2014;():

2. Moselakgomo, V. K.; Monyeki, M. A.; Toriola, A. L. Relationship between physical activity and risk factors of body weight disorders among South African primary school children. Biomedical Research-India 2015;26(4):730-738

3. Appiah, E. J.; Moses, M. O.; Alhaji, M.; Baffour-Awuah, B.; Asamoah, B.; Akwa, L. G.; Osei, F. Physiological, anthropometric profiles and motor performance of urban and rural primary school pupils. Gazzetta Medica Italiana Archivio Per Le Scienze Mediche May 2019;178(5):249-255

4. Binda, M. P.; Lukuni, M. L.; Mbensa, M. L.; Ngoma, M. Z. Interet de la mesure de la pression arterielle ambulatoire chez l'enfant suspect d'hypertension arterielle en milieu tropical. Congo méd 1993/00 1993;():733-735

5. Isaacson, C. Pathology of a Black African population. Current Topics in Pathology 1982;72():1-152

6. Nkeh-Chungag, B. N.; Chungag, A.; Sewani-Rusike, C. R. Urban and rural differences in exposure and effects of micro air particles on blood pressure parameters. Hypertension. Conference: American Heart Association's Joint Hypertension 2018;72(Supplement 1):

7. Bamigboye-Taiwo, O. T.; Ajose, O. A.; Ogunlade, O. Assessment of Risk Factors for Metabolic Syndrome in Adolescents with Obesity in Ile-Ife, South West Nigeria. West African Journal of Medicine Oct 2020;37(5):560-568

8. Damorou, F.; Yayehd, K.; Pessinaba, S.; Baragou, R.; Soussou, B. Ischemic cardiomyopathy in Lome: epidemiologic aspects and risk factors (study of 461 cases). Le Mali medical 2008;23(3)():47-54

9. Aremou, M.; Glele Ahanhanzo, G.; Gouthon, P. Comparaison des facteurs de risque atherogene entre des joueuses de handball et des non sportives. Cardiol. trop 2005/00 2005;Vol 31(123):35-39

10. Uchenwa-Onyenegecha, T. A.; Gabriel-Job, N. Hypertension and Pre-Hypertension among Children and Adolescents in Port Harcourt, Nigeria. West African Journal of Medicine Jul 29 2021;38(7):661-666

11. Daniels, Q. O.; Ukiri, R.; Lawson, F.; Lawson, L. Pattern and outcome of cardiovascular disease among children presenting at Zankli medical centre, Abuja, Nigeria. Cardiovascular Journal of Africa May-June 2011;1)():S6

12. Alakija, W. A pilot study of blood pressure levels in Benin City, Nigeria. East African Medical Journal Apr 1979;56(4):182-7

13. Monyeki, K. D.; Kemper, H. C.; Kengne, A. P. Association between body mass index, birth weight and blood pressure among rural South African children over time (1999 to 2003): Ellisras longitudinal study. Global Heart June 2016;1)():e122

14. Ajayi, S. O.; Adeoye, A. M.; Raji, Y. R.; Tayo, B.; Salako, B. L.; Ogunniyi, A.; Ojo, A.; Cooper, R. Self-reported Sleep Disorder and Ambulatory Blood Pressure Phenotypes in Patients with or without Chronic Kidney Disease: Findings from Ibadan CRECKID Study. West African Journal of Medicine Jan-Apr 2019;36(1):61-68

15. Togbe, E. K.; Annani-Akollor, M. F.; Prempeh, F. N. Obesity and co-morbidities among school children in Kumasi, Ghana. Clinical Chemistry 2017;63(Supplement 1)():S160
