# Supplementary material for: Hypertension among adolescents in sub-Saharan Africa: a systematic review
Source: Front Cardiovasc Med. 2023 Dec 7;10:1251817. doi: 10.3389/fcvm.2023.1251817 (PMC10754047; doi:10.3389/fcvm.2023.1251817)
Supplement: Supplementary file 3 [file Datasheet3.pdf]

Summary of guidelines used for defining hypertension in published research among adolescents aged 10 – 19 years in SSA

Guideline	Year	Remarks
National Heart, Lung and Blood Institute (NHLBI) Task Force Report on Blood Pressure Control in Children ***(First Report)***	1977	• Children and adolescents (up to 19 years)<br>• **Hypertension (HTN)** – Blood pressure (BP) ≥ 95th percentile for age and sex<br>• First reference population (9283)
NHLBI Report of the Second Task Force on Blood Pressure Control in Children ***(Second Report*)**	1987	• Children and adolescents (up to 18 years)<br>• "**High normal BP**" was first introduced, BP between the 90th and 95th percentile for age and sex.<br>• Hypertension was further classified into **significant HTN** (SBP or DBP between the 95th and 99th percentile for age and sex) and **severe HTN** (SBP or DBP at or above the 99th percentile)<br>• Indicated need for repeat BP measurements to confirm a diagnosis<br>• Reference population increased to 74,429<br>• Advocated for accurate identification of children with hypertension, investigation to identify children with secondary hypertension
NLBI Update on the 1987 Task Force Report on High Blood Pressure in Children and Adolescents (***Third Report)***	1996	• Children and adolescents (up to 18 years)<br>• Provided details on the relationship between height and BP.<br>• BP percentile distribution by age, sex, and **height**
NHLBI Task Force ***Fourth Report*** on the Diagnosis, Evaluation, and Treatment of High Blood Pressure in Children and Adolescents	2004	• Children and adolescents (up to 17 years)<br>• "**Prehypertension**" was introduced to replace "high normal BP"<br>• **Automated BP** devices are recommended. If BP measurement is high using an automated device, auscultatory methods should beware used for confirmation<br>• **HTN** further classified into Stage 1 (BP ≥95th and ≤ 99th percentile + 5mmHg) and Stage 2 (BP > 99th percentile + 5mmHg)
Ambulatory blood pressure monitoring in children and adolescents: Recommendations for standard assessment	2008	• Children and adolescents (up to 17 years)<br>• **White coat hypertension**: >95th percentile (office BP) + < 95th percentile (mean ABPM) + < 25% (SBP load)<br>• **Masked hypertension**: <95th percentile (office BP) + >95th percentile (mean ABPM) + > 25% (SBP load)<br>• **Prehypertension**: >95th percentile (office BP) + <95th percentile (mean ABPM) + 25% to 50% (Systolic load)<br>• **HTN**: >95th percentile (office BP) + >95th percentile (mean ABPM) + 25% to 50% (Systolic load)<br>• **Severe HTN**: >95th percentile (office BP) + >95th percentile (mean ABPM) + >50% (Systolic load)
European Society of Hypertension (**ESH**) - Management of high blood pressure in children and adolescents	2009	• Children and adolescents (up to 17 years)<br>• **High BP (Prehypertension and HTN)** defined the same as the criteria for the ***2004 Fourth Report***<br>• **High normal BP:** SBP and/or DBP ≥ 120/80 mmHg even if below the 90th percentile
American Heart Association (**AHA**) – Update - A Scientific Statement on Ambulatory BP Monitoring in Children and Adolescents	2014	• Children and adolescents (up to 17 years)<br>• **White coat HTN**: SBP and/or DBP ≥95th percentile (office BP) + < 95th percentile (mean ABPM) + < 25% (SBP or DBP load)<br>• **Prehypertension**: SBP and/or DBP ≥95th percentile or 120/80mmHg (office BP) + <95th percentile (mean ABPM) + ≥25% (SBP or DBP load)

		• **Masked HTN**: SBP and/or DBP <95th percentile (office BP) + >95th percentile (mean ABPM) + ≥ 25% (SBP or DBP load)<br>• **HTN**: SBP and/or DBP >95th percentile (office BP) + >95th percentile (mean ABPM) + 25% to 50% (SBP or DBP load)<br>• **Severe hypertension**: SBP and/or DBP >95th percentile (office BP) + >95th percentile (mean ABPM) + >50% (SBP or DBP load)
European Society of Hypertension (**ESH**) - management of high blood pressure in children and adolescents	2016	• Children and adolescents (up to 15 years)<br>• **High BP (Prehypertension and HTN)**: SBP and/or DBP ≥ 90th percentile (up to 15 years) or ≥130/85mmHg (if 16years and above)
American Academy of Paediatrics (**AAP**) - Clinical Practice Guideline for Screening and Management of High Blood Pressure in Children and Adolescents	2017	• Children and adolescents (up to 12 years)<br>• **Elevated BP** was introduced to replace prehypertension.<br>• **Obese children excluded** from the reference population<br>• The role of 24hr-ABPM for confirmation was emphasized<br>• **Elevated BP**: SBP and/or DBP ≥90th percentile and < 95th percentile OR ≥ 120/80 mmHg to <95th percentile (whichever is lower)<br>• **HTN**: SBP and/or DBP 95th percentile to ≥ 95th percentile +12 mmHg OR ≥ 140/90mmHg (whichever is lower)<br>• From 13 years, children/adolescent guideline is aligned to the guideline for adult thresholds (AHA 2017)
Hypertension guidelines for adults which are sometimes used in studies of adolescents		
World Health Organization-International Society of Hypertension Guidelines for the Management of Hypertension (**WHO – ISH**)	1999	• Adults ≥ 18 years<br>• **High normal BP:** SBP/DBP ≥ 130/85mmHg<br>• **HTN:** SBP/DBP ≥ 140/90mmHg
The Joint National Committee on Prevention, Detection, Evaluation, and Treatment of High Blood Pressure – the "*Seventh Report*" (**JNC 7**)	2003	• Adults ≥ 18 years<br>• Introduced **"prehypertension":** SBP/DBP ≥ 120/80mmHg<br>• **HTN:** SBP/DBP ≥ 140/90 mmHg
The International Diabetes Federation (**IDF**) Consensus Definition Metabolic Syndrome	2005	• Adults ≥ 18 years<br>• **High BP (Prehypertension and HTN):** SBP/DBP ≥ 130/85 mmHg
Guideline for the Management of High Blood Pressure in Adults – Eighth Joint National Committee (**JNC 8**)	2014	• Adults ≥ 18 years<br>• **HTN:** SBP/DBP ≥ 140/90 mmHg
American Heart Association (**AHA**) Guideline for the Prevention, Detection, Evaluation, and Management of High Blood Pressure in Adults	2017	• Adults ≥ 13 years<br>• **Elevated BP:** SBP/DBP ≥ 120/<80 mmHg to 129/<80 mmHg<br>• **HTN:** SBP/DBP ≥ 130/80 mmHg
